# Modeling parasite clearance and transmission delays in American Cutaneous Leishmaniasis transmission dynamics

**DOI:** 10.1007/s00285-026-02460-9

**Published:** 2026-09-18

**Authors:** Sharmin Sultana, Gilberto Gonzalez-Parra, Luis Fernando Chaves

**Affiliations:** 1 Department of Environmental and Occupational Health, School of Public Health-Bloomington, Indiana University, 2719 E. Tenth Street, Bloomington, IN 47408 USA; 2 Department of Mathematics, New Mexico Institute of Mining and Technology, 801 Leroy Pl., Socorro, NM 10587 USA; 3 Department of Geography, Indiana University, 701 E Kirkwood Ave, Bloomington, IN 47405 USA

**Keywords:** Sand flies, *Leishmania panamensis*, Hopf bifurcation, Oscillations, 34L02, 34K02, 92C60, 34D20, 92D40

## Abstract

American Cutaneous Leishmaniasis (ACL) is a multi-host vector-borne disease with complex transmission dynamics involving sand fly vectors, reservoirs, and incidental hosts. In this study, we develop and analyze two delay differential equation (DDE) models to explore the role of biological time delays in ACL transmission dynamics. The first model incorporates a delay in the parasite clearance term of the incidental host, reflecting the minimum parasite clearance time between infection and recovery. The second model introduces a delay that represents delays in skin lesion development following exposure to infective sand fly bites. For both models, we derived the basic reproduction number $\mathcal {R}_0$ and performed an elasticity analysis which revealed that vector-reservoir transmission and vector mortality are key drivers of outbreak potential. For both models we studied the stability of the disease-free and endemic equilibria. Using linearization, we identified critical delay thresholds that generate Hopf bifurcations. We show that time delays can destabilize the endemic equilibrium and induce periodic oscillations. Despite having the same $\mathcal {R}_0$, the two models exhibit distinct qualitative behaviors due to differences in the delay structure. Simulations support the analytical findings, illustrating how increasing delays can lead to oscillations in transmission. These findings highlight the importance of incorporating biologically inspired delays when modeling ACL transmission.

## Introduction

American Cutaneous Leishmaniasis (ACL) is a neglected vector-borne tropical disease caused by protozoan *Leishmania* spp. parasites, transmitted through the bites of infected sand fly (Diptera: Psychodidae) vectors (Alvar et al. 2012; Reithinger et al. 2007). ACL is endemic across rural areas of Latin America, with thousands of cases reported annually (Chaves and Pascual 2006). The disease, when associated with infections by *Leishmania panamensis*, *Leishmania mexicana* and *Leishmania braziliensis*, manifests as skin lesions that can lead to severe disfigurement, secondary infections by other pathogens, and long-term psychological consequences if left untreated (Murray et al. 2005; Saldaña 2013). ACL transmission dynamics are complex, involving interactions among sand flies, wildlife (e.g., rodents, sloths and opossums) and domestic (e.g., horses, donkeys, mules) reservoir hosts, and incidental hosts (e.g., humans), creating challenges for disease control and elimination. These challenges (González et al. 2015; Christensen et al. 1983; Aguilar et al. 1984; Chaves and Hernandez 2004; Hotez et al. 2016; Añez et al. 2018) have the potential to be more efficiently addressed, while also serving the health needs of affected populations (Chaves et al. 2024), if interventions to reduce transmission are guided by mathematical modelling.

Mathematical modeling has been widely used to understand the spread of infectious diseases, predict outbreaks, and evaluate control measures (Anderson and May 1991). Compartmental models, particularly those based on the Susceptible-Infected-Recovered (SIR) framework, have been instrumental in studying vector-borne diseases such as malaria, dengue, and leishmaniasis (Van den Driessche and Watmough 2002). These models describe disease transmission dynamics using ordinary differential equations (ODEs) and estimate key epidemiological parameters, such as the basic reproduction number ($\mathcal {R}_0$), which determines whether an infection will persist or disappear (Diekmann et al. 1990). However, many traditional models assume instantaneous transitions between disease states, overlooking biologically relevant time delays associated with incubation periods, immune responses, and latent infections (Blythe and Castillo-Chavez 1989; Grassly and Fraser 2008).

Several studies have demonstrated that incorporating delay differential equations (DDEs) into epidemiological models can significantly alter disease behavior, especially in vector-borne diseases where time-dependent processes influence transmission (Cui et al. 2019; Cooke and Van Den Driessche 1982; Saade et al. 2024, 2025; Ghosh et al. 2022). Time delays can introduce oscillatory behavior, including periodic outbreaks, and system destabilization, leading to complex epidemic patterns that differ from classical ODE-based predictions (Hethcote and Van den Driessche 2000; Liang and Shu 2020). In the case of Old World Cutaneous Leishmaniasis, periodicity in transmission has been modeled as arising from seasonality in sand fly abundance (Bacaër and Guernaoui 2006). Although such seasonal periodicity has been observed in Latin America (Feliciangeli and Rabinovich 1998; Feliciangeli 1987; Chaves et al. 2013, 2014), and is captured in time series of cases (Chaves 2009; Chaves and Pascual 2007) there is the possibility that periodicities in transmission also arise from the delay with which cutaneous lesions develop in humans, which in principle might indicate a delay in cutaneous lesion development in host populations exposed to infective sand fly bites (Chaves 2009). Similarly, infections can have a highly variable and long persistence, as suggested by parasite circulation in patients decades after the exposure to *Leishmania* spp. parasite infections (Guevara et al. 1994, 1993). Delayed infection progression can contribute to disease persistence in both human and animal populations, making ACL control and elimination efforts more difficult (Gomes et al. 2017). Understanding the role of such delays is essential for the design and evaluation of effective control measures, including vector management (Chaves et al. 2013), reservoir management, and also for improved treatment strategies (Länger et al. 2012; De Almeida and Moreira 2007).

Our early work modeling ACL transmission (Chaves and Hernandez 2004; Chaves et al. 2007, 2008b) provided insight into the importance of having vertebrate hosts (reservoirs) able to transmit pathogens to uninfected sand fly vectors and (incidental) hosts unable to transmit such pathogens when bitten by uninfected vectors. Here, we expand our framework by incorporating time delays observed in cutaneous lesion development (Chaves 2009), and delays in parasite clearance (Guevara et al. 1994, 1993), which might influence endemic stability and outbreak dynamics when incorporated into DDE models. Here, we propose two new models that capture the delays observed in ACL transmission. In the first model we explore the delay in parasite clearance, while in the second we explore the delay in ACL cutaneous lesion development after exposure to infected bites. These models allowed us to analyze how variations in fixed time delays (τ) affect the stability of the disease-free and endemic equilibria. We also explored how time delays in transmission and parasite clearance can lead to Hopf bifurcations and oscillations in incidental host population infections. More specifically, these are our main objectives for each of the two proposed DDE ACL transmission models:

- Compute $\mathcal {R}_0$ and perform an elasticity analysis of this transmission threshold.
- Investigate the stability of disease-free and endemic equilibria.
- Derive critical delays ($\tau _c$), using perturbation methods, to quantify the amplitude of oscillatory periodic solutions near the Hopf bifurcation point.
- Validate theoretical findings with numerical simulations, based on parameters estimated from field observations, and interpret biological implications for ACL transmission.

We found the two models have a common expression for the basic reproduction number. However, these DDE models exhibit different dynamical behaviors that reflect their distinct delay structures. Beyond ACL, our analysis offers broader insights into transmission dynamics driven by delays in other vector-borne and neglected tropical diseases (Massad and Coutinho 2012; Perkins et al. 2015).

## Mathematical models of ACL transmission: common assumptions and transmission thresholds

### ACL transmission biology and modeling assumptions

To construct biologically meaningful models, we assume the following(Chaves and Hernandez 2004; Chaves et al. 2007, 2008b):

- The total incidental host (*A*), reservoir host (*B*), and vector (*C*) populations are constant. This is done assuming transmission dynamics occur at a time scale where mortality from vertebrate hosts is negligible compared to the one of vectors (μ), and there is no additional recruitment of vertebrate hosts via births or migration, keeping the reservoir and the incidental host populations constant (Chaves and Hernandez 2004; Chaves et al. 2008b). For vectors, the recruitment of new individuals balances the lost due to mortality keeping the population size at the fixed value of *C* (Chaves and Hernandez 2004)
- Infected incidental hosts (*H*(*t*)) do not contribute to further transmission, meaning only infected reservoir hosts (*R*(*t*)) and infected vectors (*V*(*t*)) have a dynamic transmission feedback. This is based on the transmission biology of cutaneous leishmaniasis, where some infected vertebrate hosts, e.g., humans, are unable to infect uninfected sand fly vectors (Chaves et al. 2007). This might ultimately reflect low parasitic loads in vertebrate host species that are incidental hosts (Cecílio et al. 2022).
- To keep a reduced and tractable state space we make susceptible populations equal to uninfected individuals, i.e., $S_H(t)=A-H(t)$, $S_R(t)=B-R(t)$, $S_V(t)=C-V(t)$
- Infected reservoir hosts clear their parasitic infections at a rate $\lambda _R$, while incidental hosts clear parasites at a rate $\lambda _H$. Following parasite clearance, both reservoirs and incidental hosts can become infected again (Chaves and Hernandez 2004).
- Transmission occurs through vector bites, where a susceptible host ($S_H(t)$ or $S_R(t)$) becomes infected upon contact with an infectious vector (*V*(*t*)), or an unifected vector ($S_V(t)$) becomes infected by biting an infected reservoir (*R*(*t*)). This is the basic act of transmission in ACL (Christensen et al. 1983; Serafim et al. 2021). Transmission occurs at rates $\beta _R$, when occurring between reservoirs and vectors, and $\beta _H$, when occurring between incidental hosts and vectors.
- The system follows the mass-action principle, meaning that the infection rate is proportional to the interactions between infected and uninfected hosts and vectors (Chaves and Hernandez 2004; Calzada et al. 2015; Hethcote and Van den Driessche 2000). This is to account for the possibility of transmission once any infected vector feeds on an uninfected host (incidental and reservoir), or an unifected vector feeds on an infected reservoir host. Note that this generates an asymmetry as infected incidental hosts (*H*(*t*)) are unable to infect vectors, the original justification to have an ACL transmission model with a expanded state space (Chaves and Hernandez 2004) when compared with the classical Ross-MacDonald model for Vector-Borne Diseases (Anderson and May 1991).

Then, a basic ACL can be described by the following system of ordinary differential equations (Chaves and Hernandez 2004):

1 $$\begin{aligned} \begin{array}{crl} H'(t)& =& \beta _H \, V(t)\, (A-H(t))-\gamma _H \, H(t),\\ R'(t)& =& \beta _R V(t) \, (B-R(t))-\gamma _R \, R(t),\\ V'(t)& =& \beta _R \, R(t) (C-V(t))-\mu \, V(t), \end{array} \end{aligned}$$

### Computation of the basic reproduction number

The basic reproduction number ($\mathcal {R}_0$) is a key parameter in epidemiological models, representing the expected number of secondary infections produced by a single infected individual in a fully susceptible population (Diekmann et al. 1990). If $\mathcal {R}_0>1$, the disease can spread and persist in the population, whereas if $\mathcal {R}_0<1$, the disease will eventually die out (Dietz 1993). For diseases where transmission occurs through bloodsucking insect vectors $\mathcal {R}_0$ has a similar interpretation (Hethcote and Van den Driessche 2000; Van den Driessche and Watmough 2002). To calculate the basic reproduction number $\mathcal {R}_0$, we employ the next generation operator (*NGO*) approach (Lopez et al. 2002; Van den Driessche and Watmough 2002). When delays (τ) are added to any state variable (H(t-τ), R(t-τ), V(t-τ)) in model (1), the NGO can be computed when τ=0. Following the method described in (Lopez et al. 2002) model (1) can be linearised and written as the difference of a positive matrix (*F*), that measures how organisms get infected by a pathogen:

$$\begin{aligned} F=\begin{bmatrix} 0 & 0 & 0 \\ 0 & 0 & \beta _R \, C \\ \beta _H \, A & \beta _R \, B & 0 \end{bmatrix}. \end{aligned}$$

and a positive diagonal matrix (*T*), that measures how organisms clear pathogenic infections:

$$\begin{aligned} T=\begin{bmatrix} \gamma _H & 0 & 0 \\ 0 & \gamma _R & 0 \\ 0 & 0 & \mu \end{bmatrix}. \end{aligned}$$

The *NGO* is defined as (Diekmann et al. 1990; Van den Driessche and Watmough 2002):

$$\begin{aligned} NGO = F T^{-1}, \end{aligned}$$

where *F* is the transmission matrix and $$ T^{-1}=\begin{bmatrix} \frac{1}{\gamma _H} & 0 & 0 \\ 0 & \frac{1}{\gamma _R} & 0 \\ 0 & 0 & \frac{1}{\mu } \end{bmatrix} $$ is the inverse of *T*.

Then the *NGO* is (Lopez et al. 2002):

$$ NGO = \begin{bmatrix} 0 & 0 & \frac{\beta _H A }{\mu } \\ 0 & 0 & \frac{\beta _R B}{\mu } \\ 0 & \frac{\beta _R C }{\gamma _R} & 0 \end{bmatrix}. $$

Where the basic reproduction number $(\mathcal {R}_0)$ is the dominant eigenvalue of the matrix *NGO*, which is (Chaves and Hernandez 2004):

$$\begin{aligned} \mathcal {R}_0=\sqrt{{\frac{BC}{\mu \, \gamma _{R}}}}\beta _{R}. \end{aligned}$$

**Remark:** Note that $\mathcal {R}_0$ does not depend on $\beta _H$. Thus, reducing the value of $\beta _H$ is not crucial for the extinction of the disease. However, $\beta _H$ affects the value of *H* in the endemic state and, regardless of the value of $\beta _H$, the components of the endemic state are positive. In addition, note that $\gamma _H$ has no influence on $\mathcal {R}_0$. However, in Sect. 3.3 we will see that $\gamma _H$ has influence on the discrete time delay $\tau _c$ where the Hopf bifurcation emerges. Specifically, larger $\gamma _H$ values require a smaller the critical delay $\tau _c$ for a Hopf bifurcation to occur. Also note the $\mathcal {R}_0$ result is general for the ODE model presented in (1) and for DDE models with similar structure where any of the states variables, i.e., *H*(*t*), *R*(*t*) and/or *V*(*t*), has a fixed delay τ, as is the case for model (3) to be presented in Sect. 3 and model (62) in Sect. 4.

### Elasticity analysis of $\mathcal {R}_0$

Using the partial derivative method (Caswell 1978), we can understand how changes in parameter values can alter $\mathcal {R}_0$. We derived analytical expressions for the relative sensitivity of coefficients ($S_X$), which quantify the rate of change of $\mathcal {R}_0$ with respect to each parameter. We will use the following Normalized Sensitivity Index (Chitnis et al. 2008):

2 $$\begin{aligned} S_X=\frac{\partial \mathcal {R}_0}{\partial X} \frac{X}{\mathcal {R}_0}. \end{aligned}$$

This would give a proportional change interpretation, which has been referred in the ecological literature as elasticity (de Kroon et al. 1986). Table 1 presents the sensitivity coefficients for $\mathcal {R}_0$. Comparison of elasticities suggests that ($\beta _R$) has the greatest impact on $\mathcal {R}_0$, with a 10% increase in $\beta _R$ leading to a 10% increase in $\mathcal {R}_0$. This suggests that controlling transmission through vector management is crucial for reducing ACL transmission (Chaves et al. 2013). The populations of reservoir hosts (*B*) and vectors (*C*) also play a role, but their impact is moderate, as a 10% increase in either results in only a 5% increase in $\mathcal {R}_0$. On the other hand, the recovery rate of reservoir hosts ($\gamma _R$) and the vector mortality rate (μ) have strong negative effects on $\mathcal {R}_0$, meaning that increasing either parameter by 10% reduces $\mathcal {R}_0$ a 5%. This suggests that accelerating parasite clearance in reservoirs and incidental hosts, either through medical treatment (Añez et al. 2018) or immunotherapy (Convit et al. 1987), can reduce transmission. A similar reduction could be observed with a vector control method that increases vector mortality (Alexander and Maroli 2003).

**Table 1:** Elasticity coefficients $S_X$ with regard to $\mathcal {R}_0$ and their interpretation

Param.	$S_X$	Interpretation
A	$S_A=0$	Incidental host population does not affect $\mathcal {R}_0$
B	$S_B=\frac{1}{2}$	Increasing reservoir hosts increases $\mathcal {R}_0$
C	$S_C=\frac{1}{2}$	Increasing vector population increases $\mathcal {R}_0$
$\beta _H$	$S_{\beta _H}=0$	Incidental host transmission does not affect $\mathcal {R}_0$
$\beta _R$	$S_{\beta _R}=1$	A 10% increase in β increases $\mathcal {R}_0$ by 10%
$\gamma _H$	$S_{\gamma _H}=0$	Recovery rate of incidental hosts does not affect $\mathcal {R}_0$
$\gamma _R$	$S_{\gamma _R}=-\frac{1}{2}$	Increasing $\gamma _R$ decreases $\mathcal {R}_0$
μ	$S_\mu =-\frac{1}{2}$	Increasing μ decreases $\mathcal {R}_0$

## Modeling delays in parasite clearance

Here we present a model that includes a fixed time delay in *Leishmania* spp parasite clearance (or host recovery). In particular, reflecting the minimum parasite clearance time between infection and recovery (Chen-Charpentier 2023; Hethcote 2000), which in ACL has been reported to last from a few months (2 to 5 (Chaves 2009)) up to a couple of decades in some cases (Guevara et al. 1994, 1993). In this model, we add a fixed delay (τ) to recovering hosts in the model presented in the previous section (Chaves and Hernandez 2004). The resulting delay differential equation (DDE) model is:

3 $$\begin{aligned} \begin{array}{crl} H'(t)& =& \beta _H \, V(t)\, (A-H(t))-\gamma _H \, H(t-\tau ),\\ R'(t)& =& \beta _R V(t) \, (B-R(t))-\gamma _R \, R(t),\\ V'(t)& =& \beta _R \, R(t) (C-V(t))-\mu \, V(t), \end{array} \end{aligned}$$

Biologically, the delay (τ) represents the prolonged time to clear parasitic infections in humans as incidental hosts of ACL (Guevara et al. 1994, 1993; Saldaña 2013).

### Analysis of solutions in the delayed model *I*

The system governing the state variables *V*(*t*) and *R*(*t*) is

4 $$\begin{aligned} \frac{dR}{dt}&= \beta _R V (B - R) - \gamma _R R, \end{aligned}$$

5 $$\begin{aligned} \frac{dV}{dt}&= \beta _R R (C - V) - \mu V, \end{aligned}$$

where $\beta _R$, $\gamma _R$, μ, *B* and *C* are positive constants (parameters), and the initial conditions satisfy R(0)≥0 and V(0)≥0.

Using contradiction, we shall demonstrate that R(t)≥0 and V(t)≥0 for all t≥0.

Let us consider *V*(*t*) reaching zero for the first time at some $t_V>0$, which means

$$\begin{aligned} V(t_V)=0, \, \text {but} \, V(t)>0 \, \text {for} \, t<t_V. \end{aligned}$$

Then, at $t=t_V$, one gets

$$\begin{aligned} \frac{dV}{dt} |_{t=t_V}= \beta _R R(t_V) C. \end{aligned}$$

- If $R(t_V)>0$, then $\frac{dV}{dt} |_{t=t_V}>0$, which means *V*(*t*) is increasing at $t_V$. But this contradicts the assumption that V(t) had just reached zero for the first time.
- Similarly, if $R(t_V)<0$, then $\frac{dV}{dt} |_{t=t_V}<0$, meaning *V*(*t*) is decreasing at $t_V$. But this also contradicts the assumption that V(t) had just reached 0 for the first time.
- The only possibility left is $R(t_V)=0$.

So, we proved that when *V*(*t*) reaches zero for the first time, $R(t_V)$ must also be zero. If *V*(*t*) becomes positive again for some $t>t_V$, then

6 $$\begin{aligned} \frac{dV}{dt}=\beta _R \,R\, (C-V)-\mu V>0 \, \text { for some } \, t>t_V. \end{aligned}$$

Here, (C-V) is positive, so the term $\beta _R R (C-V)$ is positive if and only if R(t)>0. Thus for *V*(*t*) to start increasing, we must have R(t)>0.

For *R*(*t*) to become positive after $t_V$, we analyze the following equation

$$\begin{aligned} \frac{dR}{dt}= \beta _R V (B-R) -\gamma _R R. \end{aligned}$$

For *R*(*t*) to increase, we need $\frac{dR}{dt}>0$, i.e. $\beta _R V (B-R) -\gamma _R R>0$. Since we already proved that *V*(*t*) cannot increase unless R(t)>0 and we are analyzing whether *R*(*t*) can increase, this creates a circular dependency.

- *R*(*t*) can increase only if *V*(*t*) is positive.
- *V*(*t*) can increase only if *R*(*t*) is positive.

But both start at zero at $t=t_V$. Thus, neither *R*(*t*) nor *V*(*t*) can start increasing, because each depends on the other being already positive. This means that once *V*(*t*) and *R*(*t*) reach zero, they remain zero for ∀t. Analogously, if $t_R>0$ is such that

$$\begin{aligned} R(t_R)=0, \, \, \, R(t)>0 \, \, \text {for} \, \, t<t_R. \end{aligned}$$

Then, if V(0)>0 and R(0)>0, then in any case *V*(*t*) or *R*(*t*) reaches zero, they will remain at zero ∀t. Thus, neither *V*(*t*) nor *R*(*t*) have the potential to become negative.

Now consider the first equation of model (3)

$$\begin{aligned} H'(t)=\beta _H \, V(t)\, (A-H(t))-\gamma _H \, H(t-\tau ) \end{aligned}$$

with initial condition H(s)=ϕ(s)>0 for all $s \in [-\tau ,0]$, 0<H(t)≤A and $V(t) \ge \epsilon >0$ for all t≥0. If *V*(*t*) becomes small or τ becomes large, *H*(*t*) could decay to zero or become negative – even if initially positive (Singh and Ghosh 2025; Bodnar 2000; Kowalczyk and Foryś 2002).

Solving the equation of $H^{\prime }\left(t\right)$ using the integrating factor one gets

$$ H(t) = \mu (t)^{-1} \left[ H(0) + \int _0^t \mu (s) \left( \beta _H V(s) A - \gamma _H H(s - \tau ) \right) \, ds \right] $$

where the integrating factor $\mu (t)= \exp \left( \int _0^t \beta _H V(s) \, ds \right)$ is always positive and strictly increasing, since $V(t) \ge \varepsilon > 0$. Thus, if the integrand is nonnegative, then H(t)>0 for all t≥0. The extreme case for the integrand occurs when H(s-τ)=A and V(s)=infV(t). To keep *H*(*t*) nonnegative for all *s*, we require

7 $$\begin{aligned} \beta _H \cdot \inf _{t \ge 0} V(t) \cdot A \ge \gamma _H A \quad \Rightarrow \quad \beta _H \cdot \inf _{t \ge 0} V(t) \ge \gamma _H \Rightarrow \quad \inf _{t \ge 0} V(t) \ge \frac{\gamma _H}{\beta _H}. \end{aligned}$$

This gives a sufficient condition to ensure that the integrand is nonnegative and, consequently, that H(t)≥0 for all t≥0. This condition is very restrictive in scenarios of low ACL prevalence and cannot be guaranteed due to *V*(*t*)’s dependence on other state variables and parameters. Moreover, this condition is not satisfied when system (3) approaches a disease-free equilibrium point. However, there is a region in the parameter space where model (3) has positive solutions and is biologically sound. Nevertheless, finding a mathematical expression for these regions is complex, as it depends on several parameters, delays, and even the history functions (Singh and Ghosh 2025; Kowalczyk and Foryś 2002; Bodnar 2000). Here, we consider the whole parameter space to show results such as Hopf bifurcations, and biologically meaningful simulations based on parameters estimated from ACL outbreaks and research on sand fly biology (Chaves et al. 2008b).

### Steady states

The steady states of the model represent the long-term behavior of the system, where the rates of change for all state variables become zero. The time delay τ affects the dynamics, but does not directly influence the equilibria. The model admits two equilibrium points, the same for the model without lags (Chaves and Hernandez 2004):

1. Disease-Free Equilibrium (DFE):
  $$\begin{aligned} (H_0,R_0,V_0)=(0,0,0) \end{aligned}$$
  which represents a scenario in which the disease is completely absent from reservoir, incidental host and vector populations.
2. Endemic Equilibrium (EE) $E^*=(H_e, R_e, V_e)$, which exists when the basic reproduction number $\mathcal {R}_0>1$, is given by:
  $$\begin{aligned} H_e&={\dfrac{\beta _{H}\, \left( {\beta _{R}}^{2}BC-\gamma _{R}\,\mu \right) A}{\beta _{H}\, \left( {\beta _{R}}^{2}BC-\gamma _{R}\,\mu \right) +\beta _R\,\gamma _{H}\, \left( B\beta _{R}+\mu \right) }}\\ R_e&=\dfrac{\beta _R^2 B C-\gamma _R \, \mu }{\beta _{R}\, \left( \beta _{R}\,C+\gamma _{R} \right) }\\ V_e&=\dfrac{{\beta _{R}}^{2}BC-\gamma _{R}\,\mu }{\beta _{R}\, \left( B\beta _{R}+\mu \right) } \end{aligned}$$

### Disease-free equilibrium point analysis

Calculations show that system (3) has disease-free equilibrium $F^{*}_{0}=(0,0,0)$, as the system with τ=0. Linearizing the system (3) one obtains the following characteristic equation

$$ \vert J_{0} + J_{\tau }\, e^{-\lambda \tau } -\lambda I_3 \vert =0,$$

where, $$J_0=\left[ \begin{array}{ccc} 0& 0& \beta _{H}\,A\\ 0& - \gamma _{R}& B\beta _{R}\\ 0& \beta _{R}\,C& -\mu \end{array} \right] $$ and $$J_{\tau }=\left[ \begin{array}{ccc} -\gamma _{H}& 0& 0\\ 0& 0& 0\\ 0& 0& 0\end{array} \right] .$$

The characteristic equation is given by

8 $$\begin{aligned} \left( {\textrm{e}^{-\lambda \,\tau }}\gamma _{H}+\lambda \right) \left( \lambda ^2+ \left( \mu +\gamma _{R} \right) \lambda -{\beta _{R}}^{2}BC+ \gamma _{R}\,\mu \right) =0. \end{aligned}$$

The delay appears because of the accumulation of infected incidental hosts, with delayed parasite clearance (Chaves 2009), which in turn can induce periodic oscillations in the number of ACL infected incidental hosts. The characteristic equation has two eigenvalues that are the solutions of the following equation

9 $$\begin{aligned} \lambda ^2+a_1 \, \lambda +a_0=0, \end{aligned}$$

where,

$$\begin{aligned} a_1&=\mu +\gamma _R>0, \\ a_2&= \gamma _R \,\mu \, ( 1- \mathcal {R}_0), \text { with } \, \mathcal {R}_0= \sqrt{\dfrac{BC}{\gamma _R \, \mu }} \beta _R. \end{aligned}$$

We can see that $a_1$ is clearly positive and $a_2>0$ if $\mathcal {R}_0<1$. According to Routh–Hurwitz stability criterion, all roots of the polynomial in (9) have negative real parts if $a_0, \, a_1 >0$. Hence, the roots of the above quadratic equation (9) i.e. two eigenvalues of Eq. (8) have negative real parts.

The third eigenvalue is the solution of the equation

10 $$\begin{aligned} P(\lambda )={\textrm{e}^{-\lambda \,\tau }}\gamma _{H}+\lambda =0. \end{aligned}$$

To analyze stability of the above system, we consider two cases:

- Case 1: Assume λ is real.
- Case 2: Assume $\lambda =i \omega$ is purely imaginary.

**Case 1:** Solving Eq. (10) one gets

$$\begin{aligned} \lambda ={\frac{\textrm{W} \left( -\tau \,\gamma _{H}\right) }{\tau }} \end{aligned}$$

where *W* is the Lambert *W* function.

The Lambert function *W*(*x*) has multiple branches

- The principal branch $W_0(x)$ is real-valued for $x \ge - \frac{1}{\textrm{e}}$.
- The lower branch $W_{-1}(x)$ is real-valued for $-\frac{1}{\textrm{e}} \le x <0$ and complex for $x<- \frac{1}{\textrm{e}}$

Thus, one obtains that the values of λ are negative since τ and $\gamma _{H}$ are positive.

**Case 2: Hopf bifurcation analysis. ** The characteristic Eq. (8) has two main factors. To analyze a Hopf bifurcation, we specifically look for $P(\lambda )={\textrm{e}^{\lambda \,\tau }}\gamma _{H}+\lambda$ as this term is responsible for delay-induced dynamics, particularly oscillatory behavior and potential Hopf bifurcations. The second term provides non-delay stability information, which its eigenvalues must have negative real part for Hopf bifurcation to be caused solely by the delay (Guckenheimer and Holmes 2013; Marsden and McCracken 2012).

Let, $\lambda = i \omega$ (ω>0) be a root of P(λ), which is purely imaginary. Then

11 $$\begin{aligned} P(i \omega )&= \textrm{e}^{-i \omega \tau } \gamma _H + i \omega =0\end{aligned}$$

12 $$\begin{aligned}&= \left( \cos (\omega \tau ) -i\,\sin (\omega \tau ) \right) \gamma _H + i \omega =0, \end{aligned}$$

separating real and imaginary parts of the above Eq. we have

$$\begin{aligned}&\cos (\omega \tau ) \, \gamma _H=0,\\&\sin (\omega \tau ) \, \gamma _H = \omega , \end{aligned}$$

where $\cos (\omega \tau ) \, \gamma _H=0$, implies either $\cos (\omega \tau )=0$ or $\gamma _H=0$. If $\gamma _H=0$, the real part is zero, but this would imply no delay effect. If $cos(\omega \tau )=0$, then $\omega \tau = \dfrac{\pi }{2 }+n \pi$, where $n \in \mathbb {Z}$. Squaring both equations (real and imaginary parts) and then adding them, one gets $\gamma _H^2= \omega ^2$. Since ω>0, we have $\omega =\gamma _H$. Since τ>0 and ω>0 we have

13 $$\begin{aligned} \omega \, \tau =\gamma _H \, \tau = \dfrac{\pi }{2 }+n \pi , \, n=0,1,2, \dots . \end{aligned}$$

Now using $\omega =\gamma _H$ in $\sin (\omega \tau ) \, \gamma _H = \omega$, we have $\sin (\omega \tau ) = 1$. This implies,

14 $$\begin{aligned} \omega \tau =\frac{\pi }{2}+2 m \pi , \, m \in \mathbb {Z}. \end{aligned}$$

Hence the consistent solution is

15 $$\begin{aligned} \omega \tau =\frac{\pi }{2}+2 k \pi , \, k=0,1,2, \dots \end{aligned}$$

Hence, $\lambda =i \omega$ is a solution of Eq. (10) for the above values of $\omega \tau$.

**Finding a critical delay (** $\tau _c$ **)**

A Hopf bifurcation occurs when a pair of complex conjugate eigenvalues $\lambda = \pm i \omega$ cross the imaginary axis, changing the stability of the DFE (Guckenheimer and Holmes 2013; Marsden and McCracken 2012). The critical delay, $\tau _c$, is derived from the transversality condition. Using the critical frequency $\omega _c$ one gets the critical delay $\tau _c$,

16 $$\begin{aligned} \tau _c =\frac{1}{\omega _c}\left( \frac{\pi }{2}+2 k \pi \right) =\frac{1}{\gamma _H}\left( \frac{\pi }{2}+2 k \pi \right) , \, k=0,1,2, \dots \end{aligned}$$

This $\tau _c$ marks the transition point where the DFE changes from stable to oscillatory if the transversality condition is satisfied. The transversality condition ensures that the real part of at least one eigenvalue changes sign as τ crosses $\tau _c$.

Performing implicit differentiation of the characteristic equation $P(\lambda ,\tau )=0$ with respect to τ one gets:

$$\begin{aligned}&\frac{\partial P}{\partial \lambda } \frac{d \lambda }{d \tau }+ \frac{\partial P}{\partial \tau }=0,\\&\left( - \tau {\textrm{e}^{-\lambda \,\tau }}\gamma _{H}+1 \right) \frac{d \lambda }{d \tau } - \lambda {\textrm{e}^{-\lambda \,\tau }} \gamma _H=0, \\&\frac{d \lambda }{d \tau }=\frac{\lambda {\textrm{e}^{-\lambda \,\tau }} \gamma _H}{ 1- \tau {\textrm{e}^{-\lambda \,\tau }}\gamma _{H} }=\frac{\lambda \gamma _H}{ {\textrm{e}^{\lambda \,\tau }}- \tau \gamma _{H}}. \end{aligned}$$

Thus,

$$\begin{aligned} \frac{d\lambda }{d\tau }\bigg |_{\tau = \tau _c} = \frac{\gamma _H \lambda }{e^{\lambda \tau _c}-\gamma _H \tau _c}. \end{aligned}$$

We have $\gamma _H>0$ and ω>0, which implies the numerator $\gamma _H \lambda \ne 0$ because $\lambda = i\omega \ne 0$. Then substituting $\lambda =i \omega$ in the denominator one gets

$$\begin{aligned} e^{\lambda \tau _c}-\gamma _H \tau _c = \cos (\omega \tau _c) + i \sin (\omega \tau _c) -\gamma _H \tau _c. \end{aligned}$$

In a Hopf bifurcation, $\sin (\omega \tau _c) \ne 0$ (oscillatory behavior is introduced), so the denominator does not vanish. Then, the real part of $\left. \frac{d\lambda }{d\tau }\right| _{\tau =\tau _c}$ remains non-zero and the imaginary part does not dominate or cancel out the contribution of the real part. Thus, one obtains $\frac{d\lambda }{d\tau }|_{\tau =\tau _c} \ne 0$. This satisfies the transversality condition, confirming the existence of a Hopf bifurcation (Guckenheimer and Holmes 2013; Marsden and McCracken 2012). In summary, we have

- For $\tau < \tau _c$: All eigenvalues have negative real parts and the DFE is stable.
- For $\tau = \tau _c$: A Hopf bifurcation occurs, leading to periodic oscillations in the infected populations.
- For $\tau >\tau _c$: The eigenvalues $\lambda = \pm i\omega$ cross into the right half-plane, indicating instability and the onset of diverging oscillations.

**Remark:** Note that when $\mathcal {R}_0<1$ there is no endemic point (but a negative equilibrium point exists) (Jiang et al. 2009). Moreover, the DFE is locally asymptotically stable when working in $\mathbb {R}^3\ge 0$ (biological feasible region) since we can analyze the system without the equation related to *H*(*t*) and the disease becomes extinct (Jiang et al. 2009). Therefore, whenever $\mathcal {R}_0<1$ to obtain oscillations, and a Hopf bifurcation, implies that the system has negative solutions, which have no biological meaning. Thus, fully positive oscillations for model (3) cannot occur when $\mathcal {R}_0<1$.

### Stability analysis of the endemic equilibrium point $E^*$

The endemic equilibrium point in an epidemiological model represents a steady state in which the disease persists in the population (i.e., the number of infected individuals is nonzero) while the system remains in balance. It has been shown in (Chaves and Hernandez 2004) that the endemic equilibrium point is asymptotically stable when $\mathcal {R}_0>1$ and τ=0.

In this section, we analyze the existence of a Hopf bifurcation in the delayed system (3). The stability of delayed epidemic models is often analyzed using the Hopf bifurcation theory, which describes the transition from equilibrium to periodic oscillations (Kuang 1993; Marsden and McCracken 2012). Delay terms introduce time-dependent feedback into the system via H(t-τ). The Jacobian of the system at the endemic equilibrium point is given by

17 $$\begin{aligned} J_0+J_1 \, \textrm{e}^{-\lambda \,\tau }-\lambda \, I=\! \left[ \begin{array}{ccc} -\beta _{H}\,V_e-{\textrm{e}^{-\lambda \,\tau }} \gamma _{H}-\lambda & 0& \beta _{H}\, \left( A-H_e \right) \\ 0& -V_e \beta _{R}-\lambda -\gamma _{R}& \beta _{R}\, \left( B-R_e \right) \\ 0& \beta _{R}\, \left( C-V_e \right) & -R_e\beta _{R}-\lambda -\mu \end{array} \right] , \end{aligned}$$

where, $$J_{0}= \left[ \begin{array}{ccc} -\beta _{H}\,V_e& 0& \beta _{H}\, \left( A -H_e \right) \\ 0& -V_e\beta _{R}-\gamma _{R}& \beta _{R}\, \left( B-R_e \right) \\ 0& \beta _{R}\, \left( C-V_e \right) & -R_e\beta _{R}-\mu \end{array} \right] $$ and $$J_{1}= \left[ \begin{array}{ccc} -\gamma _{H}& 0& 0\\ 0 & 0& 0\\ 0& 0& 0\end{array} \right] .$$

The stability of the system is determined by the characteristic equation associated with the linearized delay differential equation (DDE):

18 $$\begin{aligned} &  P(\lambda ,\tau )({\lambda }^{2}+ \left( R_e \beta _{R}+V_e\beta _{R}+\mu +\gamma _{R} \right) \lambda -{\beta _{R}}^{2} \left( BC+BV_e+CR_e \right) \nonumber \\ &  \quad + \beta _R (R_e\,\gamma _{R}+V_e\,\mu )+\gamma _{R}\,\mu )=0 \end{aligned}$$

where $$P(\lambda ,\tau )= (e^{-\lambda \,\tau }\gamma _{H}+\beta _{H}\,V_e+\lambda ) $$.

There are two eigenvalues associated with this quadratic polynomial. These are the solutions of the following equation

19 $$\begin{aligned} &  \lambda ^2 + \left( \beta _R(V_e+R_e) + \mu + \gamma _R \right) \lambda - \beta _R^2 BC + BV_e\beta _R^2 + CR_e\beta _R^2 \nonumber \\ &  \quad + R_e\beta _R\gamma _R + V_e\beta _R\mu + \gamma _R\mu = 0, \end{aligned}$$

which can be written in the simplified form

20 $$\begin{aligned} \lambda ^2+a_1 \, \lambda +a_0=0, \end{aligned}$$

where,

21 $$\begin{aligned} a_1&=\beta _R(V_e+R_e) + \mu + \gamma _R, \end{aligned}$$

22 $$\begin{aligned} a_0&=- \beta _R^2 BC + BV_e\beta _R^2 + CR_e\beta _R^2 + R_e\beta _R\gamma _R + V_e\beta _R\mu + \gamma _R\mu . \end{aligned}$$

Substituting the values of $V_e$ and $R_e$ in $a_0$ we get

23 $$\begin{aligned} a_0=\gamma _R \mu \left( \mathcal {R}_0-1 \right) . \end{aligned}$$

Here $a_1$ is clearly positive and $a_0>0$ if $\mathcal {R}_0>1$. Considering the Routh–Hurwitz stability criterion, we have that the real part of the eigenvalues of Eq. (19) are negative.

Thus, some of the eigenvalues are the solution of the transcendental equation $P(\lambda ,\tau )=0$. Solving this for λ we have

24 $$\begin{aligned} \lambda = -\beta _H V_e + \frac{\textrm{W}\left( -\gamma _H \tau e^{\beta _H V_e \tau }\right) }{\tau }. \end{aligned}$$

Here, $\textrm{W}(.)$ represents the principal branch of the Lambert $\textrm{W}$ function. The use of the Lambert W function in the context of delay differential equations is well-documented in the literature (Corless et al. 1996; Asl and Ulsoy 2003).

If $\mathcal {R}_0>1$ the stability of the endemic equilibrium needs to be analyzed from the solutions of the following equation

25 $$\begin{aligned} {\textrm{e}^{-\lambda \,\tau }}\gamma _{H}+\beta _{H}\,V_e+\lambda =0. \end{aligned}$$

To analyze a Hopf bifurcation, we assume that the root λ takes a complex form:

$$ \lambda (\tau )=x(\tau )+i \omega (\tau ),$$

where,

- x(τ) is the real part (which determines stability),
- $\omega (\tau )$ is the imaginary part (which determines oscillations).

Given that the endemic steady state is stable when τ=0, it follows that x(0)<0. Consequently, it is important to observe that x(τ)<0 for sufficiently small τ>0, ensuring that $E^*$ remains stable. Hence, we seek to determine the critical threshold value of τ, denoted as $\tau _c$, at which a qualitative change in stability occurs. Specifically, at $\tau =\tau _c$, the eigenvalue $\lambda (\tau )$ becomes purely imaginary, implying that $x(\tau _c)=0$ while $\omega (\tau _c) \ne 0$. We have then

26 $$\begin{aligned} e^{-(x + i \omega )\tau } \gamma _H + \beta _H V_e + (x + i \omega ) = 0, \end{aligned}$$

which implies

27 $$\begin{aligned} \gamma _H e^{-x\tau } (\cos (\omega \tau ) - i\sin (\omega \tau )) + \beta _H V_e + x + i \omega = 0. \end{aligned}$$

Separating the real and imaginary parts one gets

28 $$\begin{aligned} \gamma _H e^{-x\tau } \cos (\omega \tau ) + \beta _H V_e + x = 0, \end{aligned}$$

and

29 $$\begin{aligned} -\gamma _H e^{-x\tau } \sin (\omega \tau ) + \omega = 0. \end{aligned}$$

At the Hopf bifurcation threshold, the real part of the eigenvalue must be zero (x=0). Substituting x=0 into the real and imaginary equations one gets

30 $$\begin{aligned}&\gamma _H \cos (\omega \tau _c) + \beta _H V_e = 0, \end{aligned}$$

31 $$\begin{aligned}&\gamma _H \sin (\omega \tau _c) = \omega . \end{aligned}$$

Squaring and adding the two equations to eliminate τ one gets

32 $$\begin{aligned}&\gamma _H^2 \left( \cos ^2(\omega \tau ) + \sin ^2(\omega \tau ) \right) = \beta _H^2 V_e^2 + \omega ^2, \nonumber \\&\gamma _H^2 = \beta _H^2 V_e^2 + \omega ^2, \nonumber \\&\omega = \sqrt{\gamma _H^2 - \left( \beta _H V_e \right) ^2} \, \text { as } \, \omega >0. \end{aligned}$$

The delay τ introduces a phase shift into the system through the term $\textrm{e}^{-i \omega \tau }$. For oscillations to occur, the delay τ must align with the oscillation frequency ω in a way that satisfies the phase condition of the characteristic equation. Specifically, the phase condition ensures that the oscillations repeat correctly over time (Kuang 1993; Hale and Verduyn Lunel 1993).

Now, from Eq. (31), we have $\sin (\omega \tau )= \frac{\omega }{\gamma _H}$. Since ω>0 and $\gamma _H >0$, the equation has real solutions when $0 \le \frac{\omega }{\gamma _H} \le 1$. Thus, solutions exist only when $\gamma _H \ge \omega$. The equation for $\omega \tau$ is given by

$$ \omega \tau = \arcsin \left( \frac{\omega }{\gamma _H} \right) . $$

Since sine is periodic, the general solutions for $\omega \tau$ are

$$ \omega \tau = \arcsin \left( \frac{\omega }{\gamma _H} \right) + 2k\pi , \quad k \in \mathbb {Z}. $$

where *k* represents different periodic solutions. The smallest positive solution for τ, when k=0, is:

$$\begin{aligned} \tau = \frac{1}{\omega } \arcsin \left( \frac{\omega }{\gamma _H} \right) \end{aligned}$$

The general periodic solutions are as follows:

$$ \tau = \frac{1}{\omega } \arcsin \left( \frac{\omega }{\gamma _H} \right) + \frac{2k\pi }{\omega }, \quad k \in \mathbb {Z}. $$

We have that $\cos (\omega \tau )=-\frac{\beta _H V_e}{\gamma _H}$. This has multiple solutions due to the periodicity of cosine:

33 $$\begin{aligned} \omega \tau = \pm \arccos \left( - \frac{\beta _H V_e}{\gamma _H}\right) + 2 n \pi , \; n \in \mathbb {Z}. \end{aligned}$$

Here, ω is the oscillation frequency (how fast the system oscillates in radians per unit time), $\omega \tau$ represents the total phase shift caused by the delay, and $\frac{(2n+1)\pi }{2}$ accounts for odd multiples of $\frac{\pi }{2}$ to align with the sine and cosine oscillations (Hale and Verduyn Lunel 1993).

Now, we introduce the critical delay $\tau _c$ at which a Hopf bifurcation occurs, marking the transition from stability to oscillatory dynamics, and given by

34 $$\begin{aligned} \tau _c = \frac{\pm \arccos \left( - \frac{\beta _H V_e}{\gamma _H}\right) + 2 n \pi }{\omega _c}, \; n \in \mathbb {Z}. \end{aligned}$$

where, $$\omega _c = \sqrt{\gamma _H^2 - \left( \beta _H V_e \right) ^2}$$.

**Remark:** Note that $\mathcal {R}_0$ does not depend on $\gamma _H$. Thus, reducing the value of $\gamma _H$ is not crucial for the extinction of the disease. However, $\gamma _H$ affects the value of $H^*$ in the endemic state. Moreover, $\gamma _H$ has influence on the critical delay $\tau _c$ where the Hopf bifurcation arises from the endemic state. If $\gamma _H$ becomes very small, then no Hopf bifurcation arises from the endemic state (see Eq. (34)). In fact, if $\gamma _H<\beta _H\, V_e$, then no Hopf bifurcation arises. Thus, one necessary condition for the existence of the Hopf bifurcation is $\gamma _H\ge \beta _H\, V_e$. From (7) a sufficient condition for positivity is that $\inf _{t \ge 0} V(t) \ge \frac{\gamma _H}{\beta _H}$. Then it can be deduced that $V_e$ must be equal to $\gamma _H/\beta _H$ to satisfy both conditions, but then $\omega _c$ becomes zero and $\tau _c$ becomes undefined. However, despite this, positive oscillations cannot be ruled out, since from (7) we have a sufficient condition and not a necessary condition for positivity. Thus, positive solutions can arise when $\mathcal {R}_0>1$. Nevertheless, finding a closed-form expression for the region of the parameter space where positive solutions are guaranteed is not straightforward, since it depends on several parameters, including the delay and history functions (Singh and Ghosh 2025; Bodnar 2000).

To verify the transversality condition, we proceed to compute the derivative of Re(λ) with respect to τ. For this we have the following quasi-polynomial whic is the relevant part of the characteristic equation

35 $$\begin{aligned} P(\lambda , \tau )=\gamma _H \textrm{e}^{-\lambda \tau } +\beta _H \,V_e+\lambda . \end{aligned}$$

Performing implicit differentiation of $P(\lambda ,\tau )=0$ with respect to τ one gets

$$\begin{aligned}&\frac{\partial P}{\partial \lambda } \frac{d \lambda }{d \tau }+ \frac{\partial P}{\partial \tau }=0,\\&\left( - \tau {\textrm{e}^{-\lambda \,\tau }}\gamma _{H}+1 \right) \frac{d \lambda }{d \tau } - \lambda {\textrm{e}^{-\lambda \,\tau }} \gamma _H=0, \\&\frac{d \lambda }{d \tau }=\frac{\lambda {\textrm{e}^{-\lambda \,\tau }} \gamma _H}{ 1- \tau {\textrm{e}^{-\lambda \,\tau }}\gamma _{H} }=\frac{\lambda \gamma _H}{ {\textrm{e}^{\lambda \,\tau }}- \tau \gamma _{H}}. \end{aligned}$$

At the Hopf bifurcation point, substituting $\lambda =i \omega$ one gets

36 $$\begin{aligned} \frac{d \lambda }{d \tau }&=\frac{i \omega \gamma _H}{ {\textrm{e}^{i \omega \,\tau }}- \tau \gamma _{H}} \end{aligned}$$

37 $$\begin{aligned}&=\frac{i \omega \gamma _H}{ \cos {(\omega \tau )} + i \sin {(\omega \tau )}- \tau \gamma _{H}}. \end{aligned}$$

Multiplying the numerator and denominator by the complex conjugate of the denominator one obtains

38 $$\begin{aligned} \frac{d \lambda }{d \tau }&= \frac{i \omega \gamma _H (\cos (\omega \tau )- \tau \gamma _H-i \sin (\omega \tau ))}{(\cos (\omega \tau )-\tau \gamma _H)^2+\sin ^2(\omega \tau )}\end{aligned}$$

39 $$\begin{aligned}&= \frac{i \omega \gamma _H (\cos (\omega \tau )-\tau \gamma _H)+ \omega \gamma _H \sin (\omega \tau )}{1+ \tau ^2 \gamma _H^2-2 \tau \gamma _H \cos (\omega \tau )} \end{aligned}$$

Now, separating real and imaginary parts, we have

40 $$\begin{aligned} \frac{ \omega \gamma _H \sin (\omega \tau )}{1+ \tau ^2 \gamma _H^2-2 \tau \gamma _H \cos (\omega \tau )} \end{aligned}$$

and

41 $$\begin{aligned} \frac{\omega \gamma _H (\cos (\omega \tau )-\tau \gamma _H)}{1+ \tau ^2 \gamma _H^2-2 \tau \gamma _H \cos (\omega \tau )}. \end{aligned}$$

Hence, we have

42 $$\begin{aligned} \frac{d Re(\lambda )}{d \tau } = \frac{\omega \,\gamma _H \sin (\omega \tau )}{1+ \tau ^2 \gamma _H^2-2 \tau \gamma _H \cos (\omega \tau )}. \end{aligned}$$

Recall that we obtained from the Hopf bifurcation threshold that $\cos (\omega \tau )= -\frac{\beta _H V_e }{\gamma _H}$ and $\sin (\omega \tau )=\frac{\omega }{\gamma _H}$, which gives

43 $$\begin{aligned} \frac{d Re(\lambda )}{d \tau } = \frac{ \omega ^2}{1+ \tau ^2 \gamma _H^2+2 \tau \beta _H V_e}. \end{aligned}$$

Hence $\frac{d Re(\lambda )}{d \tau }>0$ (for $\mathcal {R}_0>1$) , suggesting the Hopf bifurcation is supercritical (stable oscillations arise) (Marsden and McCracken 2012). Beyond the goals of this study, we are aware that to rigorously prove the Hopf bifurcation is supercritical would require the use of manifold theory, and analyzing the sign of the first Lyapunov exponent (Marsden and McCracken 2012; Balázs and Röst 2020). However, using numerical simulations of system (3) we can show supercritical Hopf bifurcations, since increasing the delay τ destabilizes the equilibrium point and a periodic solution arises. Moreover, it is expected that the phase-space representation of the model would converge to a periodic orbit depicting a stable limit cycle.

Now we will proceed to evaluate $\frac{d Re(\lambda )}{d \tau }$ at $\lambda =i \omega$. Using the same argument as given for the DFE $\frac{d Re(\lambda )}{d \tau } \ne 0$, we obtain the following

- When $\tau < \tau _c$, all eigenvalues have negative real parts, and the endemic equilibrium is locally asymptotically stable.
- When $\tau = \tau _c$, a pair of purely imaginary eigenvalues appears, and the system is at the brink of oscillatory behavior.
- When $\tau > \tau _c$, the real part of at least one eigenvalue becomes positive, and the system becomes unstable, undergoing to permanent oscillations.

This analysis highlights how the delay τ acts as a bifurcation parameter, determining whether the system remains at a steady state or enters a regime of periodic dynamics. Note that while $\tau < \tau _c$ ensures local asymptotic stability of the endemic equilibrium, it does not necessarily guarantee the positivity of *H*(*t*). The positivity of *H*(*t*) also depends on the balance between the infection term $\beta _H V(t)(A - H(t))$ and the loss term $\gamma _H H(t - \tau )$. Earlier analysis established that if $\inf _{t \ge 0} V(t) \ge \frac{\gamma _H}{\beta _H}$, then the infection term is strong enough to keep *H*(*t*) strictly positive for all t≥0. Therefore, delay-induced stability does not automatically ensure biological feasibility in terms of positivity - additional conditions are required in the vector population *V*(*t*). A comprehensive review on the positivity of solutions in delay differential equations is presented in Singh and Ghosh (2025). Biologically, it is also important to ensure the oscillations for *H*(*t*) don’t have values above *A*, since interpretations for H(t)>A would make model assumptions (presented in Sect. 2.1) meaningless. For example, when delays are included in the logistic density dependence population growth model, the population carrying capacity, which is often denoted as *K*, can not be interpreted as the maximum population size, as it is the case for the logistic model without delays (Vandermeer and Goldberg 2013), and instead becomes an average value around which the population oscillates (May 1974).

### Amplitude of the periodic solutions

To gain analytical insight into the behavior of the system near the Hopf bifurcation point, we consider a simplified scalar delay differential equation that captures the essential feedback dynamics between infection pressure and recovery with delay. Specifically, we isolate the equation that governs the host population *H*(*t*), and assume that the vector population *V*(*t*) remains near its quasi-steady state value $V^*$. This reduction allows us to focus on the delay-induced instability while keeping the analysis tractable (Hassard et al. 1981; Verdugo 2023, 2016).

To find the amplitude of the limit cycles we use a technique based on the Poincare-Lindstedt’s perturbation method. The perturbation uses a general expansion of order *n*, but when combining equations and doing and doing mathematical computations, the lower term that remains in a second order ODE is the cubic term. Thus, we introduce a weak cubic nonlinear damping term $-\lambda H^3(t)$, which is not present in the original biological model but is commonly employed in perturbation analysis (e.g., Hassard et al. 1981; Verdugo 2023). This term plays a crucial role in stabilizing the amplitude of oscillations beyond the bifurcation point and allows for the derivation of an explicit amplitude–delay relationship. The resulting scalar equation is given by:

44 $$\begin{aligned} \frac{dH}{dt} = \beta _H V^*(A - H(t)) - \gamma _H H(t - \tau ) - \lambda H^3(t). \end{aligned}$$

We expand around the endemic equilibrium $H^*$ by letting:

45 $$\begin{aligned} H(t) = H^* + \varepsilon h(t), \quad 0 < \varepsilon \ll 1. \end{aligned}$$

Next, we linearize the equation and include a cubic nonlinearity

$$\begin{aligned} \frac{dh}{dt}&= -a h(t) - b h(t - \tau ) + \text {nonlinear terms}, \end{aligned}$$

where $a = \beta _H V^*$, and $b = \gamma _H$. Differentiating both sides, one gets

$$\begin{aligned} \frac{d^2h}{dt^2}+ a \frac{dh}{dt} +b \frac{d h(t-\tau )}{dt}&= \varepsilon \mathcal {N}[h, h(t-\tau )], \end{aligned}$$

where $\mathcal {N}$ denotes a nonlinear term (e.g. from Taylor expansion of A-H(t)).

To accommodate a frequency shift due to weak nonlinearities, we introduce a rescaled time variable:

46 $$\begin{aligned} \theta = \Omega t, \quad \text {where } \Omega = \omega _c + \varepsilon ^2 k. \end{aligned}$$

We also expand the delay parameter as:

47 $$\begin{aligned} \tau = \tau _c + \varepsilon ^2 \delta . \end{aligned}$$

Expanding u in powers of ε, one obtains

$$ u(\theta ) = u_0(\theta ) + \varepsilon u_1(\theta ) + \cdots , $$

Then, we expand the perturbation *h*(*t*) as:

48 $$\begin{aligned} h(t) = \varepsilon u_0(\theta ) + \varepsilon ^2 u_1(\theta ) + \cdots . \end{aligned}$$

Note that while *t* is the physical time variable, all dynamics are now expressed in terms of the rescaled time θ, which evolves slightly faster or slower depending on the sign and magnitude of *k*. This change of variables is crucial: it allows each term in the expansion to remain bounded and periodic, thus ensuring the validity of the approximation over long time intervals. Now, we proceed to substitute it in the second-order equation and collect terms on the order of ε.

At $\mathcal {O}(\varepsilon ^0)$, we obtain the following

49 $$\begin{aligned} \frac{d^2 u_0}{d\theta ^2} + \omega _c^2 u_0 = 0, \end{aligned}$$

whose general solution is given by

50 $$\begin{aligned} u_0(\theta ) = A \cos (\omega _c \theta ). \end{aligned}$$

Here, *A* is the unknown amplitude to be determined.

At $\mathcal {O}(\varepsilon ^1)$, one gets

51 $$\begin{aligned} \frac{d^2 u_1}{d\theta ^2} + \omega _c^2 u_1 = F(\theta ), \end{aligned}$$

where F(θ) contains the contributions of the delay and nonlinearity. We expand the delayed argument as follows

52 $$\begin{aligned} h(t - \tau ) = \varepsilon u_0(\theta - \Omega \tau ) + \cdots \approx \varepsilon A \cos (\omega _c \theta - \omega _c \tau _c). \end{aligned}$$

For the nonlinear term, we write:

53 $$\begin{aligned} H^3(t) = (H^* + \varepsilon u_0)^3 = \cdots + \varepsilon ^3 u_0^3 + \cdots , \end{aligned}$$

and assume $\lambda = \lambda _1 / \varepsilon ^2$, giving

54 $$\begin{aligned} \lambda H^3(t) \approx -\lambda _1 u_0^3. \end{aligned}$$

Using the identity $\cos ^3(x) = \frac{3}{4} \cos (x) + \frac{1}{4} \cos (3x)$, we obtain that

55 $$\begin{aligned} u_0^3(\theta ) = A^3 \cos ^3(\omega _c \theta ) = \frac{3}{4} A^3 \cos (\omega _c \theta ) + \frac{1}{4} A^3 \cos (3\omega _c \theta ). \end{aligned}$$

Thus, the forcing term becomes

56 $$\begin{aligned} F(\theta ) = - \gamma _H A \cos (\omega _c \theta - \omega _c \tau _c) -\frac{3}{4} \lambda _1 A^3 \cos (\omega _c \theta ) -\frac{1}{4} \lambda _1 A^3 \cos (3\omega _c \theta ). \end{aligned}$$

To eliminate secular growth in $u_1(\theta )$, the coefficient of $\cos (\omega _c \theta )$ in F(θ) must vanish. Using the identity:

57 $$\begin{aligned} \cos (\omega _c \theta - \omega _c \tau _c) = \cos (\omega _c \theta ) \cos (\omega _c \tau _c) + \sin (\omega _c \theta ) \sin (\omega _c \tau _c), \end{aligned}$$

we isolate the resonant part:

58 $$\begin{aligned} \gamma _H A \cos (\omega _c \tau _c) \cos (\omega _c \theta ) - \frac{3}{4} \lambda _1 A^3 \cos (\omega _c \theta ). \end{aligned}$$

Setting the coefficient of $\cos (\omega _c \theta )$ to zero, we obtain that

59 $$\begin{aligned} \gamma _H A \cos (\omega _c \tau _c) - \frac{3}{4} \lambda _1 A^3 = 0. \end{aligned}$$

Solving for $A^2$ gives:

60 $$\begin{aligned} A^2 = \frac{4 \gamma _H \cos (\omega _c \tau _c)}{3 \lambda _1}. \end{aligned}$$

Near the bifurcation, we approximate $\cos (\omega _c \tau _c) \approx b (\tau - \tau _c)$, yielding the final expression of the amplitude:

61 $$\begin{aligned} A^2 = \frac{4 \omega _c^2 b}{3 \lambda _1} (\tau - \tau _c)=\frac{4 \omega _c^2 \gamma _H}{3 \lambda _1} \Delta . \end{aligned}$$

**Fig. 1:**
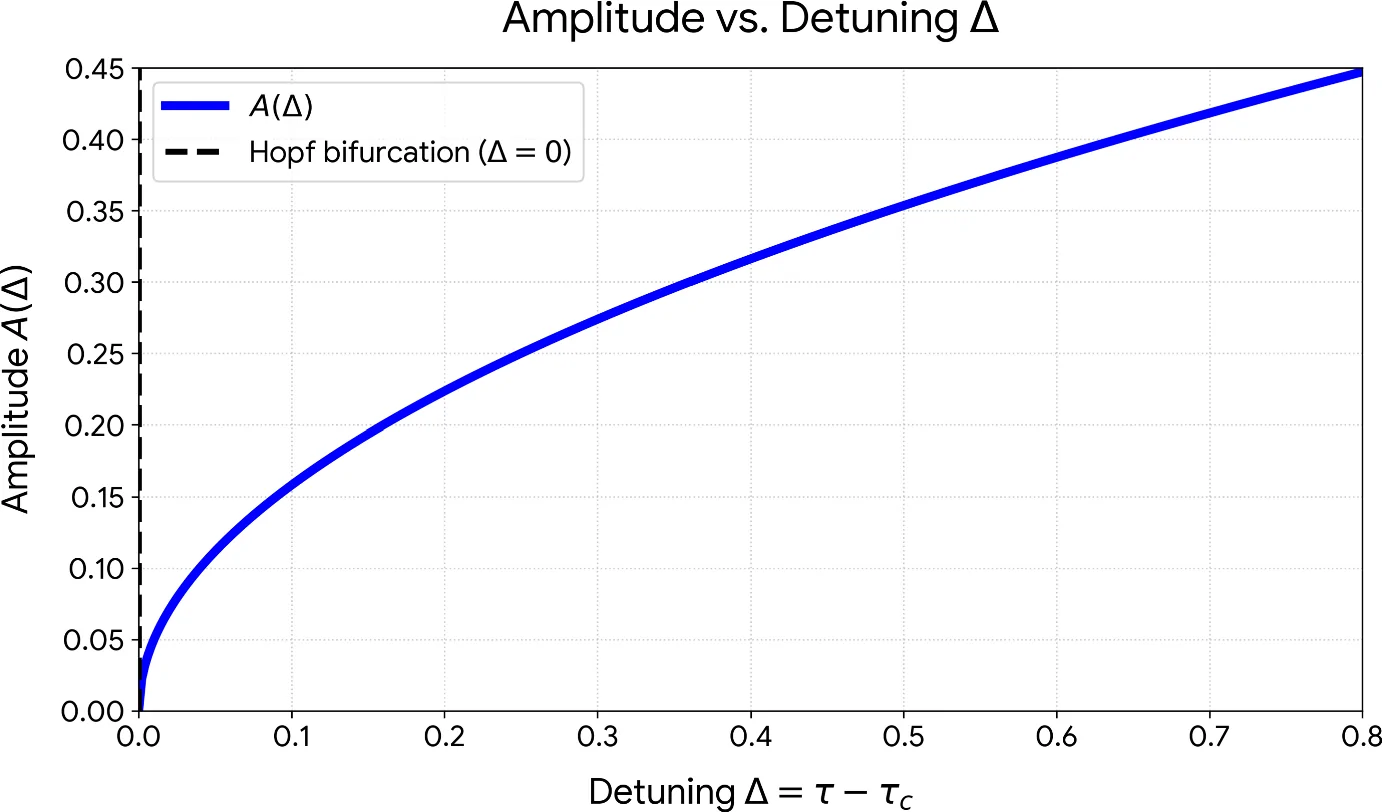
Amplitude of the periodic solution as a function of the detuning parameter $\Delta = \tau - \tau _c$. The amplitude grows as $\sqrt{\Delta }$, consistent with a supercritical Hopf bifurcation

This expression confirms that the amplitude of the periodic solution grows proportionally to $\sqrt{\Delta }$, which is characteristic of a supercritical Hopf bifurcation (see Guckenheimer 2008). (Fig. 1)

### Numerical simulations

In this section, we numerically explore the dynamics of the ACL model (3), focusing on the effects of the time delay on the system’s stability and behavior. We rely on the MATLAB 2024 built-in function dde23 which provides numerical solutions for systems of delay differential equations (Shampine and Thompson 2001). For the simulations we vary the transmission rate $\beta _R$ in order to have scenarios with the basic reproduction number below and above one. Other parameters are based on relevant field and laboratory studies (Chaves et al. 2008b). This provides additional support for the theoretical analysis, where $\mathcal {R}_0$ is a threshold for qualitative differences in model dynamics.

#### Numerical simulations and analysis when $\mathcal {R}_0<1$

**Fig. 2:**
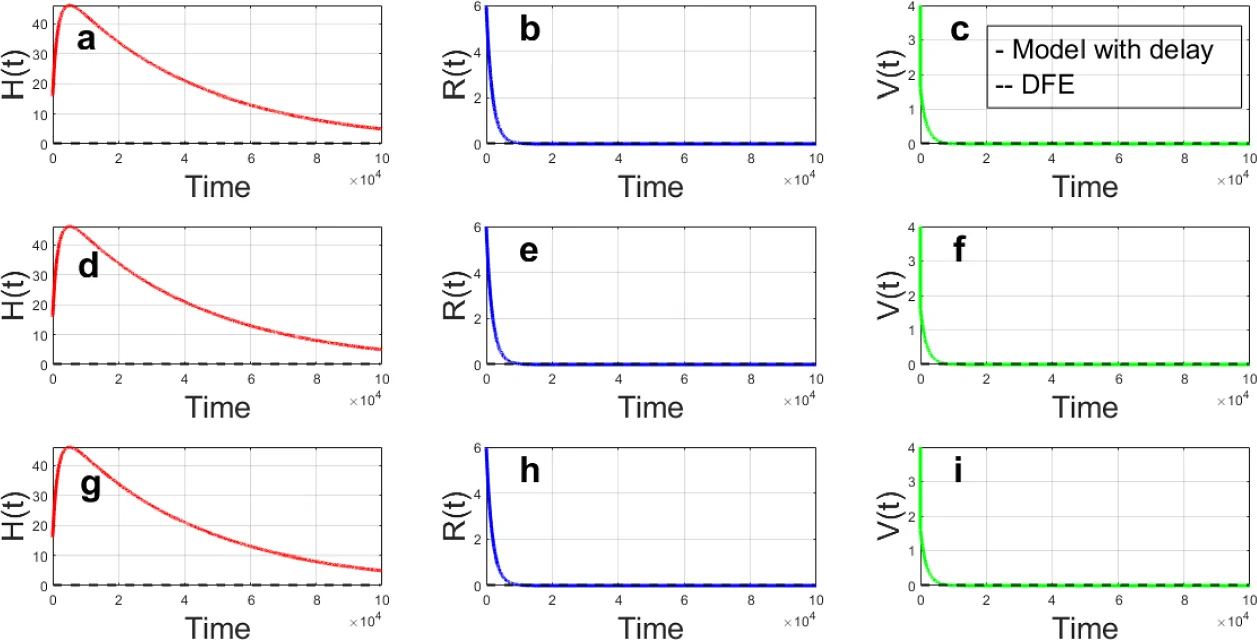
Numerical simulations of ACL model (3) for different delays (τ) when when $\mathcal {R}_0 < 1$. Panels **a**, **b** and **c** show the dynamics when τ=1. Panels **d**, **e** and **f** show the dynamics when τ=2 and panels **g**, **h** and **i** show the dynamics when τ=3. In all simulations $\mathcal {R}_0=0.5$, $\beta _H = 0.000139, \, \beta _R = 0.000023369, \, \gamma _H = 0.000024, \, \gamma _R = 0.0007, \, \mu = 0.42, \, A = 124, \, B = 28,$ and C=4807. Initial conditions are $H(0)=16, \, R(0)=6, \, V(0)=4$. Solid lines in panels **a**, **d** and **g** show the dynamics for incidental hosts. Dotted portions of the lines indicate biologically unrealistic negative values. Panels **b**, **e** and **h** show the dynamics for reservoirs, panels **c**, **f** and **i** show the dynamics for vectors. DFE indicates the disease free equilibrium, represented by a dashed line in each panel. Parameters estimates for $\gamma _H$, $\gamma _R$, μ and initial are from (Chaves et al. 2008b) which are based on a field study (Aguilar et al. 1984)

As we showed in section 2.3 when $\mathcal {R}_0<1$ no biologically meaningful oscillations are possible, as oscillations will include negative values for *H*(*t*). In Fig. 2 we illustrate the transmission dynamics for *H*, *R* and *V* for three different values of delay τ. We reduce $\gamma _H$ and the delays to show biologically feasible solutions (positive), that also represent infinitely many positive solutions in a parameter space of model (3) when $\mathcal {R}_0<1$ (Guckenheimer and Holmes 2013; Marsden and McCracken 2012; Bodnar 2000).

#### Numerical simulations and analysis when $\mathcal {R}_0>1$

**Fig. 3:**
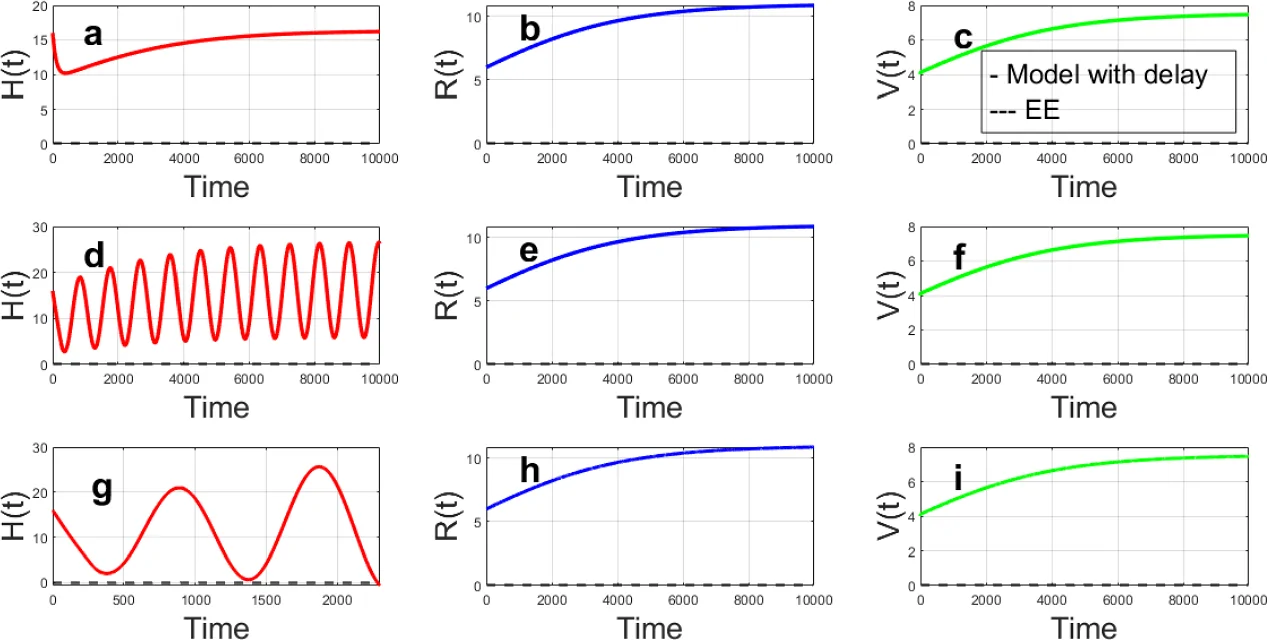
Numerical simulations of model (3) for different delays when $\mathcal {R}_0 > 1$. In all simulations $\mathcal {R}_0=1.2838$, $\beta _H = 0.000141, \, \beta _R = 0.00006, \, \gamma _H = 0.007, \, \gamma _R = 0.0007, \, \mu = 0.42, \, A = 124, \, B = 28$, and C=4807. Initial conditions are $H(0)=16, \, R(0)=6, \, V(0)=4$. Solid lines in panels **a**, **d** and **g** show the dynamics for incidental hosts. Panels **b**, **e** and **h** show the dynamics for reservoirs, panels **c**, **f** and **i** show the dynamics for vectors. Top, middle and bottom rows show the solutions with $\tau \approx 0.09\tau _c, \tau \approx \tau _c$ and $\tau \approx 1.19\tau _c$ respectively. The critical delay is $\tau _c\approx 227$. EE indicates the expected endemic equilibrium, represented by a dashed line in each panel. Parameters estimates are from (Chaves et al. 2008b) which are based on a field study (Aguilar et al. 1984)

In this section, we perform numerical simulations of the mathematical model (3) for different values of the time delay τ when $\mathcal {R}_0>1$. Figure 3 presents the dynamics of *H*, *R* and *V* for different values of the delay parameter τ, when $\mathcal {R}_0\approx 1.28$, which is based on modeling (Chaves et al. 2008b) data from a major field study on ACL transmission in Venezuela (Aguilar et al. 1984).

**Case 1:** $\tau < \tau _c$ (Stable Endemic Equilibrium).

Figure 3 (top row) shows that the solution converges to the endemic equilibrium $E^*$. The system stabilizes at a constant endemic level.

**Case 2:** $\tau = \tau _c$ (Onset of Hopf bifurcation).

Figure 3 (middle row) shows emerging oscillations in *H*(*t*). This represents a Hopf bifurcation in which the endemic equilibrium transitions from a stable state to periodic oscillations. The population *H* oscillates, forming a limit cycle and thus supporting the theoretical results. The reservoir host *R*(*t*) and vector *V*(*t*) populations remain positive throughout the simulation and converge to the endemic equilibrium $E^*$.

**Case 3:** $\tau > \tau _c$ (Instability and divergence)

Figure 3 (bottom row) shows that *H*(*t*) has oscillations that grow larger or diverge, indicating instability in the system. Indeed, *H*(*t*) takes negative values. On the other hand, the populations *R*(*t*) and *V*(*t*) remain positive throughout the simulation and converge to the endemic equilibrium.

**Fig. 4:**
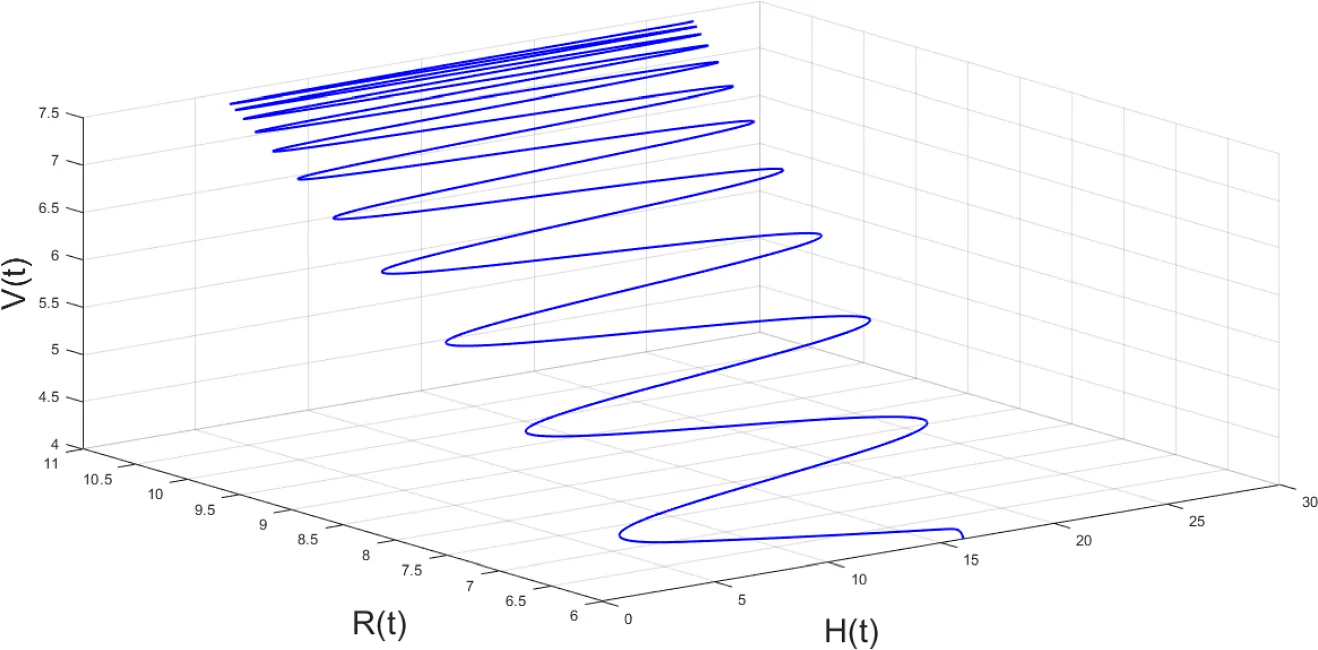
Numerical solution of model (3) in the phase space for the critical delay when $\mathcal {R}_0 > 1$. In all simulations $\mathcal {R}_0=1.2838$, $\beta _H = 0.000141, \, \beta _R = 0.00006, \, \gamma _H = 0.007, \, \gamma _R = 0.0007, \, \mu = 0.42, \, A = 124, \, B = 28$, and C=4807. Initial conditions are $H(0)=16, \, R(0)=6, \, V(0)=4$

Figure 4 shows one solution in the phase space using the critical delay, which shows convergence to a periodic orbit as expected in a stable limit cycle (Vandermeer and Goldberg 2013).

## Modeling delays in transmission

A distinguishing characteristic of ACL is the non-instantaneous progression from infected bite exposure to skin lesion development in incidental hosts. After being bitten by an infected vector, parasites undergo a multiplication period before the development of skin lesions in the infected host (Chaves 2009; Saldaña 2013). This type of delay has been studied by Rafiq et al. (2022). However, that study did not derive critical delay thresholds, $\tau _c$, and did not study the potential for the existence of Hopf bifurcations and other destabilizing oscillations. Epidemiological studies report that the average delay between infection and the onset of skin lesions in ACL can range from weeks to several months, underscoring the biological necessity of accounting for this time lag, which has been observed when fitting models to time series data (Chaves 2009).

Mathematically, this biological delay introduces a memory effect into the system: the number of incidental hosts newly infected at time *t* depends on the number of infected vectors and susceptible hosts at the earlier time t-τ. This delayed feedback plays a crucial role in shaping the system’s dynamics and can lead to oscillatory behavior or instabilities that are not captured in models without delay (Agyingi and Wiandt 2019; Roy et al. 2015; Ruan 2008). Thus, in this work we consider that the new infected hosts are due to the effective contact between the number of infected vectors and susceptible hosts at the earlier time t-τ (Hethcote 2000). Therefore, we consider the following system of DDEs to represent transmission delays:

62 $$\begin{aligned} \frac{dH}{dt}&= \beta _H V(t - \tau )(A - H(t - \tau )) - \gamma _H H(t), \nonumber \\ \frac{dR}{dt}&= \beta _R V(t)(B - R(t)) - \gamma _R R(t), \nonumber \\ \frac{dV}{dt}&= \beta _R R(t)(C - V(t)) - \mu V(t) , \end{aligned}$$

where *H*(*t*), *R*(*t*), and *V*(*t*) denote the populations of infectious incidental hosts, reservoirs, and vectors, respectively. The discrete time delay τ represents the time between the exposure to bites by infected vectors and the development of a cutaneous skin lesion.

### Steady states

The model admits two biologically meaningful steady states: disease-free and endemic equilibria. The disease-free equilibrium corresponds to the complete absence of infection in all populations and is given by:

$$\begin{aligned} E_0 = (H^*, R^*, V^*) = (0, 0, 0). \end{aligned}$$

The endemic equilibrium represents persistent disease within all populations and is given by:

$$ E^* = \left( \frac{\beta _H V^* A}{\beta _H V^* + \gamma _H},\; \frac{\beta _R V^* B}{\beta _R V^* + \gamma _R},\; \frac{\beta _R^2 B C - \mu \gamma _R}{\beta _R (\beta _R B + \mu )} \right) , $$

where $V^* = \frac{\beta _R^2 B C - \mu \gamma _R}{\beta _R (\beta _R B + \mu )}$. Note that $V^*$ is positive if $\beta _R^2 B C > \mu \gamma _R$.

Since incidental hosts do not contribute to transmission, the infection is maintained through the reservoir–vector transmission loop.

### Disease-free equilibrium point analysis

Linearizing system (62) one obtains the following characteristic equation

$$ \vert J_{0} + J_{\tau }\, e^{-\lambda \tau } -\lambda I_3 \vert =0,$$

where, $$J_0=\left[ \begin{array}{ccc} -\gamma _H& 0& 0\\ 0& - \gamma _{R}& B\beta _{R}\\ 0& \beta _{R}\,C& -\mu \end{array} \right] $$ and $$J_{\tau }=\left[ \begin{array}{ccc} 0& 0& \beta _H A\\ 0& 0& 0 \\ 0& 0& 0\end{array} \right] $$.

Thus, the simplified characteristic equation is

$$\begin{aligned} \left( \gamma _{H}+\lambda \right) \left( {\lambda }^{2}+ \left( \gamma _{R}+\mu \right) \lambda +\gamma _{R}\,\mu -{\beta _{R}}^{2}BC \right) =0 \end{aligned}$$

This result is surprising because the delay is absent in the characteristic equation. From a mathematical viewpoint this occurs because the delayed term only appears in a non-leading diagonal position. Thus, the delay does not influence the characteristic equation in the DFE. From an epidemiological point of view this occurs since previous states of the vector in the DFE do not affect the particular dynamics of the host population. Therefore, one root of the characteristic equation is $\lambda =-\gamma _H<0$. The other two eigenvalues are the solutions of the following equation,

63 $$\begin{aligned} \lambda ^2+a_1 \, \lambda +a_0=0, \end{aligned}$$

which is exactly the same as Eq. (9). Using the same argument as we used for Eq. (9), we can conclude that all the eigenvalues have negative real parts if $\mathcal {R}_0<1$. As a result, the DFE is locally asymptotically stable.

### Global stability of the disease-free equilibrium

#### Theorem 1

The disease-free equilibrium point $E_0 = (0, 0, 0)$ of system (62) is globally asymptotically stable in $\mathbb {R}_{\ge 0}$ if $\mathcal {R}_0<1$. This result holds for any delay $\tau \ge 0$.

#### Proof

We construct the following Lyapunov function for the reservoir–vector subsystem:

$$ \mathcal {L}(R, V) = R + \frac{\gamma _R}{\beta _R C} V. $$

This function is non-negative for all R,V≥0 and $\mathcal {L}(R, V) = 0$ only at the DFE.

We compute the derivative along the solutions of the system:

$$ \begin{aligned} \frac{d\mathcal {L}}{dt}&= \frac{dR}{dt} + \frac{\gamma _R}{\beta _R C} \frac{dV}{dt} \\&= \left[ \beta _R V(B - R) - \gamma _R R \right] + \frac{\gamma _R}{\beta _R C} \left[ \beta _R R(C - V) - \mu V \right] \\&= \beta _R V(B - R) - \gamma _R R + \gamma _R R \left( 1 - \frac{V}{C} \right) - \frac{\gamma _R \mu }{\beta _R C} V \\&= \beta _R B V - \frac{\gamma _R \mu }{\beta _R C} V - \beta _R R V - \frac{\gamma _R R V}{C}\\&= \left( \frac{\beta _R^2 BC- \mu \gamma _R }{\beta _R C} \right) V -RV \left( \beta _R + \frac{\gamma _R}{C}\right) \\&= \frac{ \mu \gamma _R}{\beta _R C} \left( \mathcal {R}_0^2-1\right) V -RV \left( \beta _R + \frac{\gamma _R}{C}\right) . \end{aligned} $$

Now note that all terms are non-positive if $\mathcal {R}_0 \le 1$. Hence,

$$ \frac{d\mathcal {L}}{dt} \le 0, $$

with equality only at R=0, V=0. By LaSalle’s invariance principle, (R(t),V(t))→(0,0) as $t \rightarrow \infty$.

Now consider the equation for H(t),

$$ \frac{dH}{dt} = \beta _H V(t - \tau )(A - H(t - \tau )) - \gamma _H H(t). $$

Since $V(t - \tau ) \rightarrow 0$ as $t \rightarrow \infty$, for any ε>0, there exists T>0 such that $V(t-\tau )<\varepsilon$ for all t>T. Then for any t>T we get the linear differential inequality

$$\begin{aligned} \frac{dH}{dt} < \beta _H \varepsilon A-\gamma _H H(t). \end{aligned}$$

Let η(t) satisfy the linear differential equation

$$\begin{aligned} \frac{d\eta }{dt} = \beta _H \varepsilon A - \gamma _H \eta (t), \quad \eta (T) = H(T). \end{aligned}$$

Using comparison theorem, we have $H(t) \le \eta (t)$ for all t>T, and

$$ \eta (t) \rightarrow \frac{\beta _H \varepsilon A}{\gamma _H} \quad \text {as} \quad t \rightarrow \infty . $$

Since ε was arbitrary, we conclude that H(t)→0 as $t \rightarrow \infty$.

Therefore, (H(t),R(t),V(t))→(0,0,0), and the DFE is globally asymptotically stable. □

### Stability analysis of the endemic equilibrium point

Delay terms introduce time-dependent feedback into the system via H(t-τ) and V(t-τ). The Jacobian of the system at the endemic equilibrium point is given by

64 $$\begin{aligned} J_0+J_1 \, \textrm{e}^{-\lambda \,\tau }-\lambda \, I_3= \left[ \begin{array}{ccc} -\gamma _{H}-{\textrm{e}^{-\lambda \,\tau }} \beta _H V^*-\lambda & 0& \beta _{H}\, \left( A-H^* \right) {\textrm{e}^{-\lambda \,\tau }} \\ 0& - \beta _{R} V^*-\gamma _{R} -\lambda & \beta _{R}\, \left( B-R^* \right) \\ 0& \beta _{R}\, \left( C-V^*\right) & -\beta _{R} R^*-\mu -\lambda \end{array} \right] \end{aligned}$$

where, $$J_{0}= \left[ \begin{array}{ccc} -\gamma _H& 0& 0 \\ 0& -\beta _{R} V^*-\gamma _{R}& \beta _{R}\, \left( B-R^* \right) \\ 0& \beta _{R}\, \left( C-V^* \right) & -\beta _{R} R^*-\mu \end{array} \right] $$ and $$J_{1}= \left[ \begin{array}{ccc} -\beta _{H} V^*& 0& \beta _H (A-H^*)\\ 0 & 0& 0\\ 0& 0& 0\end{array} \right] $$

Then the characteristic equation of the system (62) is

$$\begin{aligned} \text {det} \left( J_0+J_1 \, \textrm{e}^{-\lambda \,\tau }-\lambda \, I_3 \right) =0 \end{aligned}$$

This is a transcendental equation involving ${\textrm{e}^{-\lambda \,\tau }}$. Due to delay, the stability of the endemic equilibrium depends on delay τ.

The simplified characteristic equation associated with the linearization around the endemic equilibrium $E^* = (H^*, R^*, V^*)$ is given by

65 $$\begin{aligned} &  \left( \lambda + \gamma _H + \beta _H V^* e^{-\lambda \tau } \right) \nonumber \\ &  \quad \left[ (\lambda + \gamma _R + \beta _R V^*)(\lambda + \mu + \beta _R R^*) - \beta _R^2 (B - R^*)(C - V^*) \right] = 0. \end{aligned}$$

One root is the solution of the following equation

66 $$\begin{aligned} \lambda + \gamma _H + \beta _H V^* e^{-\lambda \tau }=0 \end{aligned}$$

When the delay τ is small, we can approximate the exponential term using a first-order Taylor expansion:

$$ e^{-\lambda \tau } \approx 1 - \lambda \tau . $$

Substituting into the delayed equation

$$ \lambda + \gamma _H + \beta _H V^* e^{-\lambda \tau } = 0, $$

we obtain:

$$ \lambda + \gamma _H + \beta _H V^* (1 - \lambda \tau ) = 0. $$

Simplifying:

$$ \lambda (1 + \beta _H V^* \tau ) = -(\gamma _H + \beta _H V^*). $$

Solving for λ, we get:

$$ \lambda = -\frac{\gamma _H + \beta _H V^*}{1 + \beta _H V^* \tau }. $$

Since all the parameters are positive, the real part of λ is negative, for a sufficiently small delay τ.

The other two roots are the solution of the following equation

67 $$\begin{aligned} \lambda ^2 + \left( \beta _R(V^*+R^*) + \mu + \gamma _R \right) \lambda + \beta _R^2 (BV^* + CR^*-BC) +\beta _R(\gamma _R R^* + \mu V^*) + \gamma _R\mu = 0 \end{aligned}$$

which is exactly same as equation (19). From there we know that both the eigenvalues are negative. As a result, all the eigenvalues of the characteristic equation (65) are negative. Hence, the endemic equilibrium remains locally asymptotically stable for sufficiently small delay τ.

### Hopf bifurcation analysis

We analyze the existence of a Hopf bifurcation arising from the endemic equilibrium $E^* = (H^*, R^*, V^*)$ as the delay parameter τ varies. The delay appears only in the first component of the characteristic equation:

$$ \lambda + \gamma _H + \beta _H V^* e^{-\lambda \tau } = 0. $$

This is a transcendental equation whose roots determine the local stability of the endemic equilibrium. A Hopf bifurcation occurs when a pair of complex conjugate roots crosses the imaginary axis as τ increases.

Let $\lambda = i\omega$, with ω>0. Substituting into the equation gives:

$$ i \omega + \gamma _H + \beta _H V^* e^{-i \omega \tau } = 0. $$

Using Euler’s formula, $e^{-i \omega \tau } = \cos (\omega \tau ) - i \sin (\omega \tau )$, we separate real and imaginary parts:

$$\begin{aligned} \gamma _H + \beta _H V^* \cos (\omega \tau )&= 0, \\ \omega - \beta _H V^* \sin (\omega \tau )&= 0. \end{aligned}$$

Rearranging

68 $$\begin{aligned} \beta _H V^* \cos (\omega \tau )&= -\gamma _H, \end{aligned}$$

69 $$\begin{aligned} \beta _H V^* \sin (\omega \tau )&= \omega . \end{aligned}$$

Squaring and adding equations (68) and (69), we obtain:

$$ \left( \frac{\gamma _H}{\beta _H V^*} \right) ^2 + \left( \frac{\omega }{\beta _H V^*} \right) ^2 = 1 \quad \Rightarrow \quad \omega = \sqrt{(\beta _H V^*)^2 - \gamma _H^2}. $$

This yields a real value for ω if and only if $\beta _H V^* > \gamma _H$, which is the condition for a Hopf bifurcation.

From equation (68), we solve for $\omega \tau$:

$$ \cos (\omega \tau _c) = -\frac{\gamma _H}{\beta _H V^*}. $$

which implies

$$\begin{aligned} \tau _c = \frac{1}{\sqrt{(\beta _H V^*)^2 - \gamma _H^2}} \left( \cos ^{-1} \left( -\frac{\gamma _H}{\beta _H V^*} \right) + 2\pi n \right) , \quad n \in \mathbb {Z}_{\ge 0} \end{aligned}$$

So, the Hopf bifurcation occurs at

$$ \tau _c = \frac{1}{\sqrt{(\beta _H V^*)^2 - \gamma _H^2}} \left( \cos ^{-1} \left( -\frac{\gamma _H}{\beta _H V^*} \right) + 2\pi n \right) \quad \text {provided that } \beta _H V^* > \gamma _H. $$

For $\tau < \tau _c$, the endemic equilibrium is locally asymptotically stable; for $\tau > \tau _c$, the system undergoes sustained oscillations through a Hopf bifurcation.

#### Verification of the transversality condition

Consider the transcendental equation (66) arising from the linearization around the endemic equilibrium.

Assume that for some critical delay $\tau = \tau _c$, the equation has a pair of purely imaginary roots $\lambda = i\omega$, with ω>0. To verify the occurrence of a Hopf bifurcation, we must confirm that the transversality condition holds, namely:

$$ \left. \frac{d \text {Re}(\lambda )}{d\tau } \right| _{\tau = \tau _c} \ne 0. $$

We differentiate both sides of the characteristic equation implicitly with respect to τ, treating $\lambda = \lambda (\tau )$:

$$ \frac{d\lambda }{d\tau } + \beta _H V^* \frac{d}{d\tau } \left( e^{-\lambda \tau } \right) = 0. $$

we get

$$ \frac{d\lambda }{d\tau } - \beta _H V^* e^{-\lambda \tau } \left( \tau \frac{d\lambda }{d\tau } + \lambda \right) = 0. $$

Solving for $$ \frac{d\lambda }{d\tau } $$:

$$ \frac{d\lambda }{d\tau } \left( 1 - \beta _H V^* \tau e^{-\lambda \tau } \right) = \beta _H V^* \lambda e^{-\lambda \tau }, $$

$$ \Rightarrow \quad \frac{d\lambda }{d\tau } = \frac{\beta _H V^* \lambda e^{-\lambda \tau }}{1 - \beta _H V^* \tau e^{-\lambda \tau }}. $$

To evaluate the transversality condition, we let $\lambda = i\omega$, where ω>0 is the Hopf frequency. Then:

$$ e^{-\lambda \tau } = e^{-i \omega \tau } = \cos (\omega \tau ) - i \sin (\omega \tau ), $$

$$ \lambda e^{-\lambda \tau } = i \omega (\cos (\omega \tau ) - i \sin (\omega \tau )) = \omega \sin (\omega \tau ) + i \omega \cos (\omega \tau ). $$

So the numerator becomes

$$ \beta _H V^* \lambda e^{-\lambda \tau } = \beta _H V^* \omega \sin (\omega \tau ) + i \beta _H V^* \omega \cos (\omega \tau ). $$

Now the denominator becomes

$$ 1 - \beta _H V^* \tau e^{-\lambda \tau } = 1 - \beta _H V^* \tau (\cos (\omega \tau ) - i \sin (\omega \tau )) = a + ib, $$

where

$$ a = 1 - \beta _H V^* \tau \cos (\omega \tau ), \qquad b = \beta _H V^* \tau \sin (\omega \tau ). $$

Then the real part of $\dfrac{d\lambda }{d\tau }$ is given by:

$$ \text {Re} \left( \frac{d\lambda }{d\tau } \right) = \text {Re} \left( \frac{x + i y}{a + ib} \right) = \frac{xa + yb}{a^2 + b^2}, $$

where

$$ x = \beta _H V^* \omega \sin (\omega \tau ), \qquad y = \beta _H V^* \omega \cos (\omega \tau ). $$

After substitution we have

$$ \text {Re} \left( \frac{d\lambda }{d\tau } \right) = \frac{ \beta _H V^* \omega \left[ \sin (\omega \tau )(1 - \beta _H V^* \tau \cos (\omega \tau )) + \cos (\omega \tau )(\beta _H V^* \tau \sin (\omega \tau )) \right] }{ \left( 1 - \beta _H V^* \tau \cos (\omega \tau ) \right) ^2 + \left( \beta _H V^* \tau \sin (\omega \tau ) \right) ^2 }. $$

Simplifying the numerator:

$$ \sin (\omega \tau )(1 - \beta _H V^* \tau \cos (\omega \tau )) + \cos (\omega \tau )(\beta _H V^* \tau \sin (\omega \tau )) = \sin (\omega \tau ). $$

So the final expression is:

$$ \text {Re} \left( \frac{d\lambda }{d\tau } \right) = \frac{ \beta _H V^* \omega \sin (\omega \tau ) }{ \left( 1 - \beta _H V^* \tau \cos (\omega \tau ) \right) ^2 + \left( \beta _H V^* \tau \sin (\omega \tau ) \right) ^2 } $$

This expression is strictly positive under the Hopf conditions, verifying the transversality condition. Hence, a Hopf bifurcation occurs at $\tau = \tau _c$, and the endemic equilibrium loses stability through the emergence of periodic oscillations.

### Numerical simulations

In this section, we explore the dynamics of the ACL model presented in (62), focusing on the effects of the transmission time delay on the stability and transient behavior of the system.

#### Numerical simulations when $\mathcal {R}_0<1$

**Fig. 5:**
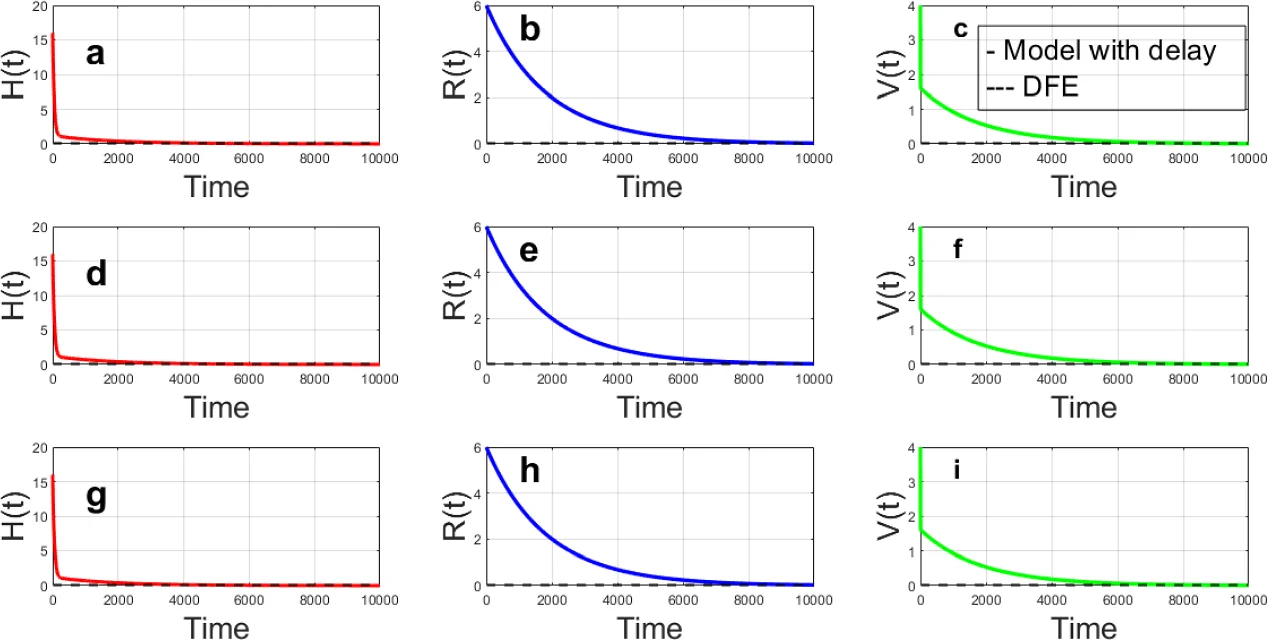
Numerical solutions of ACL model (62) for different delays (τ) when $\mathcal {R}_0 < 1$. Panels **a**, **b** and **c** show the dynamics when $\tau =40<\tau _c$. Panels **d**, **e** and **f** show the dynamics when $\tau =65.45 \approx \tau _c$ and panels **g**, **h** and **i** show the dynamics when $\tau =80>\tau _c$. In all simulations $\mathcal {R}_0=0.5$, $\beta _H = 0.000139, \, \beta _R = 0.000023369, \, \gamma _H = 0.024, \, \gamma _R = 0.0007, \, \mu = 0.42, \, A = 124, \, B = 28,$ and C=4807. Initial conditions are $H(0)=16, \, R(0)=6, \, V(0)=4$. Solid lines in panels **a**, **d** and **g** show the dynamics for incidental hosts. Panels **b**, **e** and **h** show the dynamics for reservoirs, panels **c**, **f** and **i** show the dynamics for vectors. DFE indicates the expected disease free equilibrium, represented by a dashed line in each panel. Parameters estimates are from (Chaves et al. 2008b) which are based on a field study (Aguilar et al. 1984)

Figure 5 illustrates the numerical solutions of model (62) with $\mathcal {R}_0 < 1$. The basic reproduction number was set to $\mathcal {R}_0 = 0.5$. Despite increasing the delay to τ=80, the system consistently converges to the DFE. Specifically, incidental hosts H(t), reservoirs R(t), and vectors V(t) all show a rapid exponential decay toward zero, which is consistent with the analytically predicted global stability of the DFE in the subthreshold regime.

Notably, while the delay affects the transient dynamics, including the speed of convergence and small deviations before stabilization, it does not alter the long-term behavior. In all cases, regardless of the delay, the infected populations ultimately vanish. This confirms that the delay does not affect the stability of the DFE when $\mathcal {R}_0 < 1$. These numerical results align with the theoretical analysis, which shows that the DFE remains globally asymptotically stable for all $\tau \ge 0$ in this regime.

#### Numerical simulations when $\mathcal {R}_0>1$

**Fig. 6:**
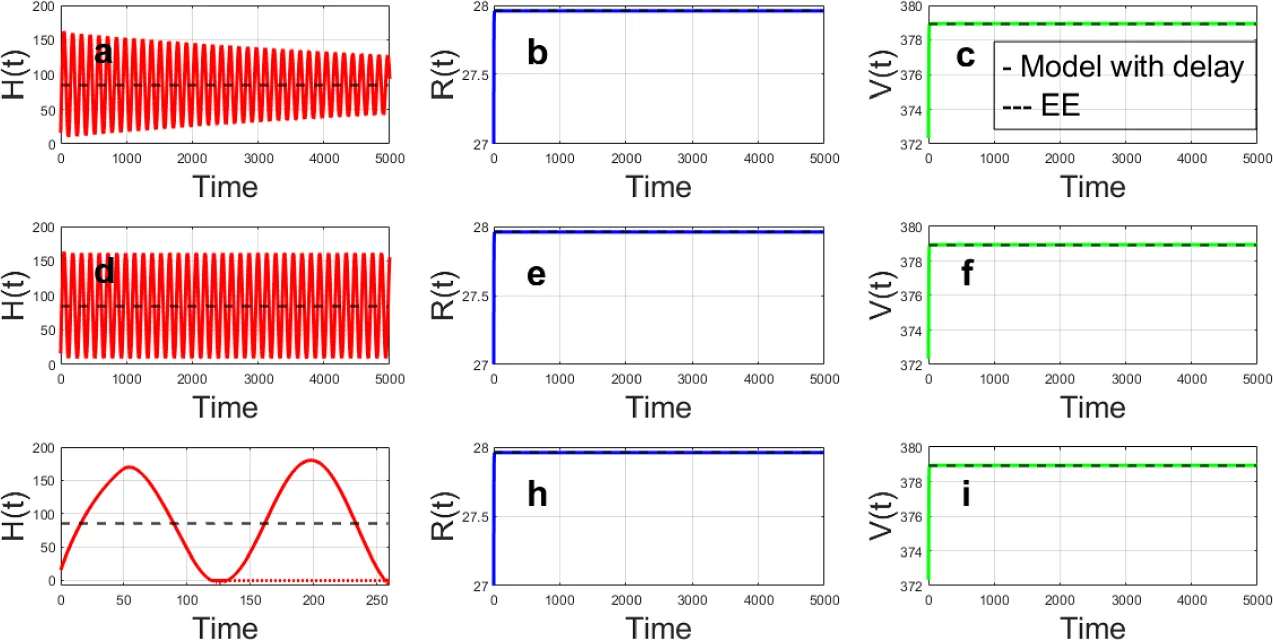
Numerical simulations of model (62) for different delays (τ) when $\mathcal {R}_0 > 1$. Panels **a**, **b** and **c** show the dynamics when $\tau \approx 0.99\tau _c$. Panels **d**, **e** and **f** show the dynamics when $\tau = \tau _c\approx 43$ and panels **g**, **h** and **i** show the dynamics when $\tau =1.1\tau _c$. In all simulations $\mathcal {R}_0\approx 27$, $\beta _H = 0.000139, \, \beta _R = 0.0128550, \, \gamma _H = 0.024, \, \gamma _R = 0.0007, \, \mu = 0.42, \, A = 124, \, B = 28,$ and C=4807. Initial conditions are $H(0)=16, \, R(0)=27, \, V(0)=374$. Solid lines in panels **a**, **d** and **g** show the dynamics for incidental hosts. Panels **b**, **e** and **h** show the dynamics for reservoirs, panels **c**, **f** and **i** show the dynamics for vectors. EE indicates the expected endemic equilibrium, represented by a dashed line in each panel. Parameters estimates are from (Chaves et al. 2008b) which are based on a field study (Aguilar et al. 1984)

In this section, we will perform numerical simulations of system (62) for different values of the time delay τ when $\mathcal {R}_0>1$. The Hopf bifurcation analysis, derived analytically earlier in this paper, predicts that this endemic state loses stability at a critical delay value $\tau _c \approx 43$. Figure 6 presents the dynamics of *H*, *R* and *V* for different values of the delay parameter τ.

**Case 1:** $\tau < \tau _c$ (Stable Endemic Equilibrium).

Figure 6 (panels a, b and c) the solution converges to the endemic equilibrium. All populations remain positive throughout the simulation.

**Case 2:** $\tau = \tau _c$ (Onset of Hopf bifurcation).

When the delay increases to the bifurcation point $\tau _c$, periodic oscillations signal a supercritical Hopf bifurcation, as small-amplitude stable periodic solutions appear around the endemic equilibrium, see Fig. 6 (panels d, e and f).

**Case 3:** $$\tau > \tau _c$$ (Instability).

In this case the oscillatory behavior becomes divergent with pronounced increases in amplitude, as can be seen in Fig. 6 (panels g, h and i).

**Fig. 7:**
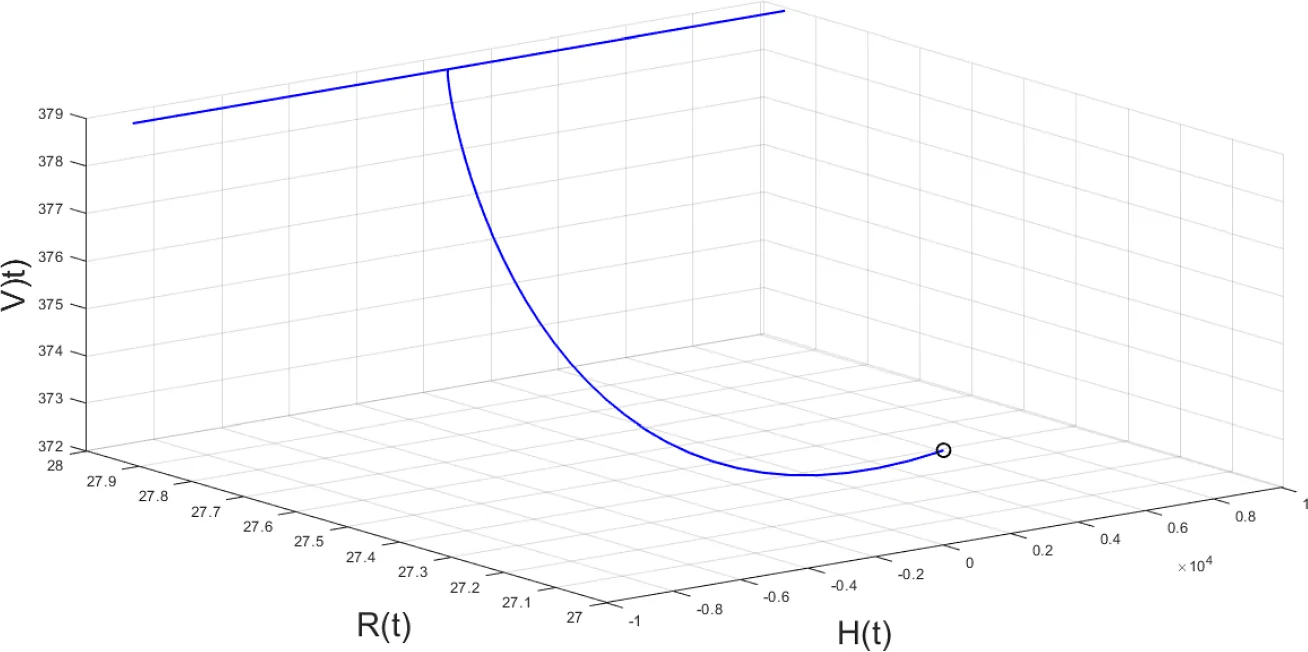
Numerical simulation of model (62) in the phase space using the critical delay $\tau _c$. In the simulation $\mathcal {R}_0\approx 27$, $\beta _H = 0.000139, \, \beta _R = 0.00128550, \, \gamma _H = 0.024, \, \gamma _R = 0.0007, \, \mu = 0.42, \, A = 124, \, B = 28,$ and C=4807. Initial conditions are $H(0)=16, \, R(0)=27, \, V(0)=374$

Figure 7 shows one solution in the phase space using the critical delay. The reservoir and vector populations become infected very fast, but the incidental host population keeps oscillating.

## Discussion

The current study, where we used parameters estimates from a field study (Chaves et al. 2008b) in model simulations, provides an opportunity to further study the transmission dynamics in the ACL outbreak from Las Rosas, Cojedes State, Venezuela (Aguilar et al. 1984). Overall, it seems the delay in transmission is more likely to be the one acting at the population level for ACL transmission. Supporting this possibility are extensive records of people developing lesions months after being exposed to sand fly bites in endemic areas (Guevara et al. 1994; Chaves 2009), and the existence of autocorrelation patterns in time series of ACL cases that do not correspond to seasonal transmission (Chaves 2009), as expected from models that consider seasonal fluctuations in sand fly populations (Bacaër and Guernaoui 2006).

We also need to consider that, as argued by Guevara et al. (1994), Guevara et al. (1993), the occurrence of extreme delays in parasite clearance seems to be extremely rare, so for modeling transmission dynamics at the population level it might be negligible. However, understanding why parasitic infections can show a long delay in creating a pathology is nevertheless a topic worth of study using models for intra-host parasite population dynamics (Länger et al. 2012), and the role that chronic infections, with low parasitic loads, could have on the absence of pathologies, as it has been described for malaria parasites (Chaves et al. 2009).

The evaluation of delays τ in relation to the critical thresholds $\tau _c$ suggests that is unlikely that transmission delays (τ) in nature are equal or larger than $\tau _c$, but it is possible they still can produce transient oscillations, that are persistent because of perturbations that keep pushing the transmission system away from its endemic equilibrium.

An open question from our results is the potential for resonance between cyclic transmission patterns that arise from the interaction of seasonal sand fly abundance fluctuations (Feliciangeli and Rabinovich 1998; Feliciangeli 1987), with the developmental delay in skin lesion generation (Añez et al. 2018; Guevara et al. 1994; Chaves 2009), which might result in the stochastic amplification of epidemics, a phenomenon that has been studied in other diseases (Alonso et al. 2007).

## Conclusions

In this study we expand a simple model that was developed to study ACL transmission dynamcis over 20 years ago (Chaves and Hernandez 2004). The first model incorporates a delay in parasite clearance of incidental hosts, whereas the second model introduces a delay in the transmission from vectors to incidental hosts. Unlike standard compartmental models that assume instantaneous transitions between disease states, both models incorporate time delays that introduce memory into the system.

Analytically, we established that both systems have a critical delay threshold $\tau _c$ beyond which a Hopf bifurcation occurs. This bifurcation marks the transition from a stable equilibrium (disease-free or endemic) to periodic oscillations, signifying recurrent outbreaks of ACL. These analytical solutions were validated through numerical simulations, which demonstrated that delays fundamentally change the long-term dynamics of the disease.

For $\mathcal {R}_0 < 1$, the two models behave differently. In the second model, the disease-free equilibrium (DFE) is globally asymptotically stable for all $\tau \ge 0$, as confirmed both analytically and numerically. In contrast, the first model can produce oscillations that are biologically unrealistic, as they include negative values for *H*(*t*)

When $\mathcal {R}_0 > 1$, small time delays τ lead the system into a stable endemic equilibrium. As τ increases toward $\tau _c$, both models exhibit the Hopf bifurcation, resulting in sustained periodic oscillations. For $\tau > \tau _c$, the systems exhibit growing oscillation amplitudes or instability, making disease control and long-term prediction increasingly difficult. Although the onset of oscillations occurs in both models, the structure and amplitude of these oscillations vary depending on the placement of the delay, illustrating how different biological mechanisms of latency influence epidemic patterns.

Understanding the mechanism behind periodicities in ACL Chaves and Pascual (2007); Chaves et al. (2014) is important to optimize the deployment of vector control methods (Chaves et al. 2013), including novel methods such as those based on bacteria that inhibit parasite multiplication in sand fly vectors (Cecilio et al. 2025), as the potential impact in reducing transmission can be robustly evaluated with the use of mathematical models with different assumptions (Qu et al. 2018, 2022).

Finally, future work can explore the role of seasonal variations and climate fluctuations in vector population abundance and infection (Feliciangeli 1987; Bacaër and Guernaoui 2006; Chaves et al. 2013, 2014). This will allow the quantification of how environmental factors interact with oscillation generating delays in shaping ACL recurrent seasonal and interannual epidemics (Chaves and Pascual 2006), including consideration for the potential role of distributed delays (Chuang et al. 2017). Further developments could include modeling the muti-scale implications of within-host immune responses (Länger et al. 2012; De Almeida and Moreira 2007) to understand population level transmission (Chaves 2009), synergisms with inapparent infections (Safan and Altheyabi 2023), host preference (Chaves et al. 2007; Caja Rivera and Barradas 2019), and nonlinearities that emerge from control that intensifies with disease burden (Sultana et al. 2026). Another important extension is developing models that connect land use land cover changes with changes in transmission (Chaves 2013; Vinson et al. 2022), based on the observations about the nonlinear association between forest cover and ACL transmission (Yamada et al. 2016), reflecting changes in sand fly fauna composition (Rigg et al. 2021) and infection (Rigg 2019), and the role that social class structure (Okamoto et al. 2022) and marginalization (Chaves et al. 2008a) have on shaping spatial and temporal patterns of ACL transmission. All these extensions would improve our understanding of ACL persistence and improve the quality of modeling insights and predictions that could serve communities when proposing and executing actions to reduce ACL transmission.

## Data Availability

No datasets were generated or analyzed during the current study.
